# Federated multimodal AI for precision-equitable diabetes care

**DOI:** 10.3389/fdgth.2025.1678047

**Published:** 2026-01-16

**Authors:** Bing Bai, Xilin Liu, Hong Li

**Affiliations:** 1 Medical Service Dimension, China-Japan Union Hospital of Jilin University, Changchun, China; 2 Department of Hand and Foot Surgery, China-Japan Union Hospital of Jilin University, Changchun, China; 3 Department of Nursing, China-Japan Union Hospital of Jilin University, Changchun, China

**Keywords:** artificial intelligence, digital-twin, federated learning, health-equity, precision medicine

## Abstract

Type 2 diabetes mellitus (T2DM) constitutes a rapidly expanding global epidemic whose societal burden is amplified by deep-rooted health inequities. Socio-economic disadvantage, minority ethnicity, low health literacy, and limited access to nutritious food or timely care disproportionately expose under-insured populations to earlier onset, poorer glycaemic control, and higher rates of cardiovascular, renal, and neurocognitive complications. Artificial intelligence (AI) is emerging as a transformative counterforce, capable of mitigating these disparities across the entire care continuum. Early detection and risk prediction have progressed from static clinical scores to dynamic machine-learning (ML) models that integrate multimodal data—electronic health records, genomics, socio-environmental variables, and wearable-derived behavioural signatures—to yield earlier and more accurate identification of high-risk individuals. Complication surveillance is being revolutionised by AI systems that screen for diabetic retinopathy with near-specialist accuracy, forecast renal function decline, and detect pre-ulcerative foot lesions through image-based deep learning, enabling timely, targeted interventions. Convergence with continuous glucose monitoring (CGM) and wearable technologies supports real-time, AI-driven glycaemic forecasting and decision support, while telemedicine platforms extend these benefits to remote or resource-constrained settings. Nevertheless, widespread implementation faces challenges of data heterogeneity, algorithmic bias against minority groups, privacy risks, and the digital divide that could paradoxically widen inequities if left unaddressed. Future directions centre on multimodal large language models, digital-twin simulations for personalised policy testing, and human-in-the-loop governance frameworks that embed ethical oversight, trauma-informed care, and community co-design. Realising AI's societal promise demands coordinated action across patients, clinicians, technologists, and policymakers to ensure solutions are not only clinically effective but also equitable, culturally attuned, and economically sustainable.

## 1 Introduction

**Figure 1:**
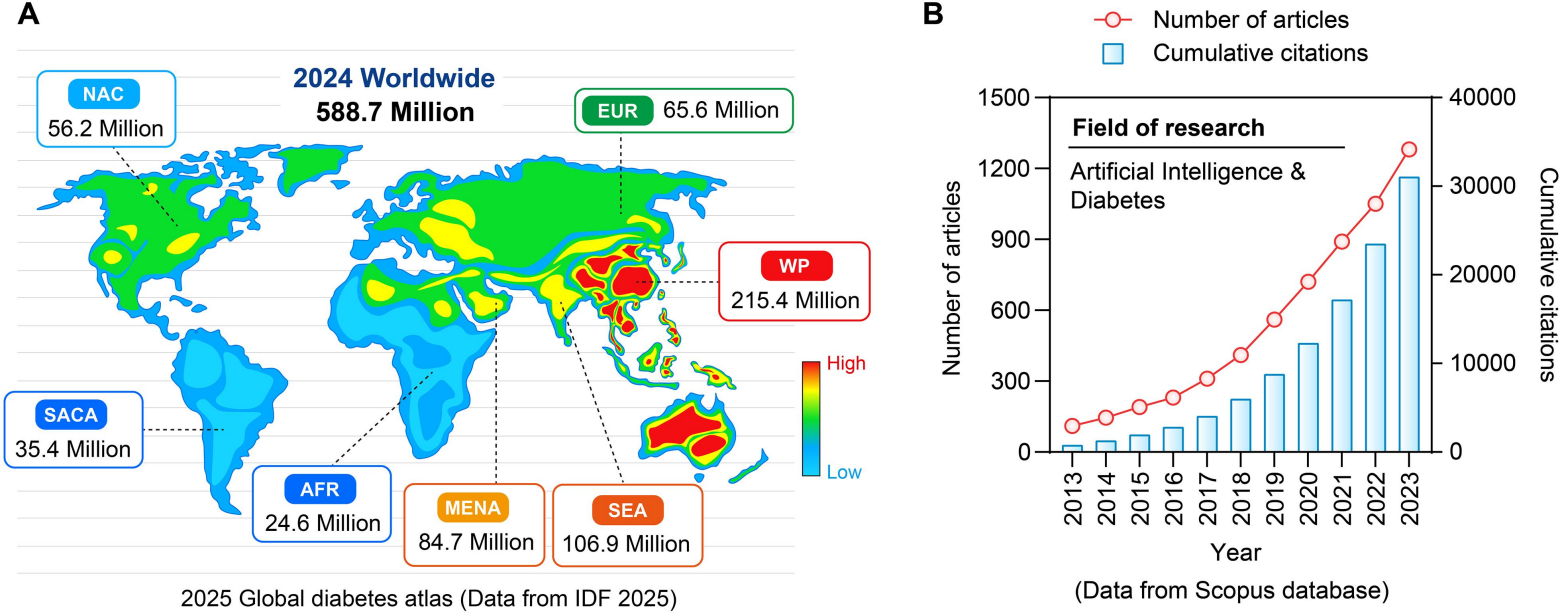
Prevalence rate of diabetes in the world and AI issuing trend. **(A)** Distribution of diabetes patients worldwide in 2024. **(B)** Research papers and citation situations in the field of “artificial intelligence and diabetes” from 2013 to 2023.

Diabetes's social burden is a complex issue with societal, psychological, and economic facets in addition to personal health consequences. T2DM, in particular, has become a global epidemic that disproportionately affects underinsured people, members of racial and ethnic minorities, and those from socioeconomically disadvantaged backgrounds (1) (Figure 1). Cardiovascular disorders, renal failure, and cognitive decline are among the complications of the disease that worsen its effects on society and put a tremendous burden on healthcare systems and economies around the world (2). Diabetes management and outcomes are greatly influenced by social determinants of health, which frequently reinforce health disparities. These factors include childhood environment, education, socioeconomic status, and access to healthcare (3). The lack of access to wholesome food, rigid healthcare schedules, and low health literacy are some of the obstacles that people from underprivileged backgrounds usually encounter when trying to manage their health effectively. These factors all work together to worsen glycemic control and increase the likelihood of complications (1, 3). The confluence of these elements emphasizes how urgently creative, scalable solutions are needed to alleviate the social burden of diabetes.

Although this is a narrative review rather than a systematic review, we adhered to a structured approach for literature selection to ensure comprehensiveness and relevance. We conducted a comprehensive search of PubMed, Web of Science, and Embase databases from inception until October 2025, using a combination of keywords and MeSH terms related to “artificial intelligence,” “machine learning,” “diabetes mellitus,” “diabetic complications,” “federated learning,” “digital twin,” and “health equity.”

Studies were included if they: (1) focused on AI/ML applications in diabetes care, including risk prediction, complication monitoring, or personalized interventions; (2) were published in peer-reviewed journals in English; (3) involved human subjects or real-world data; and (4) reported measurable outcomes (e.g., AUC, sensitivity, specificity, clinical impact).

Exclusion criteria included: (1) conference abstracts, editorials, or letters without original data; (2) studies limited to technical model development without clinical relevance; and (3) non-English publications.

After removing duplicates, titles and abstracts were screened by two independent reviewers (BB and HL), with disagreements resolved by consensus. Full texts of potentially relevant articles were assessed for eligibility. In addition, reference lists of key articles and recent high-quality reviews were manually searched to identify additional relevant studies. The final selection was based on thematic relevance, methodological quality, and representation of diverse populations and settings.

With previously unheard-of potential to lessen the disease's societal impact, AI marks a revolutionary shift in the treatment of diabetes. AI-driven technologies have shown great promise in lowering complications, increasing patient engagement, and improving glycemic control. Examples of these technologies include CGM, automated insulin delivery (AID) systems, and predictive analytics (4, 5). It has been demonstrated that AID systems help patients with type 1 and type 2 diabetes spend more time within target glucose ranges and lower their HbA1c levels, which lessens the strain of daily self-management (5, 6). Furthermore, AI-powered tools can offer real-time, personalized advice, improving treatment adherence and enabling patients to actively participate in their care (7). Nevertheless, there are certain difficulties in incorporating AI into diabetes care. Thorough monitoring and ethical considerations are required for issues like data privacy, algorithmic bias, and the danger of becoming overly dependent on technology (7, 8). In order to ensure that underprivileged groups profit from these developments, AI adoption must be equitable. The need for standardized, culturally relevant solutions is highlighted by the fact that, although AI-based diabetic retinopathy (DR) screening systems have demonstrated high negative predictive values, their sensitivity varies greatly (8).

AI's potential to alleviate the social burden of diabetes is further enhanced by its convergence with wearable technology and telemedicine. Continuous glycemic level and behavior monitoring is made possible by wearable technology, which also offers real-time feedback that can guide tailored interventions (2). AI in conjunction with social media platforms promotes a cooperative approach to diabetes management by facilitating peer-to-peer support and patient-provider interactions (2). To guarantee their accessibility and scalability, however, extensive clinical trials and cost-effectiveness evaluations are necessary for the broad adoption of these technologies (2). Social determinants of health, systemic barriers to care, and health equity must be given top priority when integrating AI into public health strategies. High-risk populations' ability to manage their diabetes can be improved by trauma-informed care models that take into consideration the psychological and emotional effects of childhood adversity (9). Healthcare professionals can customize interventions to match the specific requirements of various populations by using AI to comprehend and address the larger sociopolitical contexts that influence health behaviors (3).

Diabetes has a significant social impact that necessitates creative, comprehensive solutions. AI holds great promise for improving outcomes, reducing disparities, and lessening the disease's impact on society, making it a significant turning point in the treatment of diabetes. But for AI to be successfully incorporated into diabetes care, a well-rounded strategy that tackles moral, technical, and societal issues is needed. For AI-driven innovations to be fair, available, and efficient, cooperation between patients, healthcare professionals, technologists, and legislators is crucial. The healthcare industry can transform diabetes care and lessen its significant societal burden by embracing AI's potential while maintaining moral principles and putting patient-centered care first (8).

### 1.1 Fundamental concepts

A revolutionary approach to precision-equitable diabetes care is represented by the convergence of federated learning (FL) and multimodal artificial intelligence (AI), which tackles important issues with data privacy, interoperability, and tailored intervention (10). Diabetes mellitus (DM) is a rising global health burden that necessitates creative ways to reduce inequalities in care outcomes and access, especially for underprivileged groups (10). Traditional centralized AI models face limitations due to fragmented healthcare data and stringent privacy regulations, which hinder cross-institutional collaboration (11). Federated multimodal AI emerges as a robust framework to overcome these barriers by enabling decentralized model training across heterogeneous datasets while preserving patient confidentiality (12). FL facilitates collaborative model development without raw data exchange, aligning with privacy-preserving regulations such as HIPAA and PIPEDA (12). In diabetes care, FL has demonstrated efficacy in predicting complications and optimizing glycemic control using electronic health records from multi-province cohorts (11). Studies leveraging SecureBoost and vertical-FL have shown promise in integrating diverse clinical features across institutions sharing the same patient population but differing in feature spaces (12). This approach mitigates biases inherent in single-center datasets and enhances model generalizability (11). However, challenges persist in scenarios with label scarcity, necessitating semi-supervised FL techniques that combine labeled and unlabeled data (13). Innovations like Federated PU Learning address settings where some institutions possess only positive and unlabeled samples, while others contribute purely unlabeled data, enabling robust risk prediction even with sparse annotations (14). Multimodal AI integrates diverse data modalities—continuous glucose monitoring (CGM), insulin pump logs, activity trackers, and genomic markers—to capture the complex pathophysiology of diabetes (10, 14). Unlike unimodal models, multimodal frameworks leverage cross-modal alignment and co-learning to enhance predictive accuracy and clinical relevance (10, 15). While image-to-text translation models enhance diabetic retinopathy screening, time-series analysis of CGM data in conjunction with NLP-derived dietary logs can forecast glycemic variability (10, 14). By pretraining models on unlabeled multimodal pairs, recent developments in self-supervised learning (SSL) further reduce reliance on labeled data, enabling label-efficient fine-tuning for personalized care. Two major challenges are representation learning and fusion strategies (10, 15).

## 2 Main methodologies

### 2.1 Early screening and risk prediction

**Figure 2:**
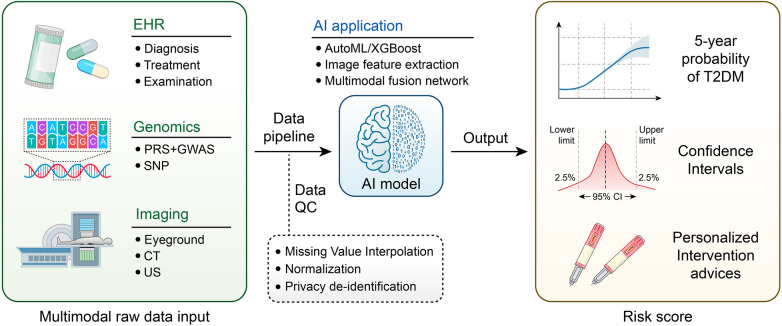
Workflow of multimodal artificial intelligence for diabetes risk prediction.

A revolutionary change in healthcare is represented by the incorporation of AI into risk prediction and early screening frameworks, especially when considering chronic illnesses and intricate clinical outcomes. Clinical decision-making has traditionally relied on traditional scoring systems, such as the Finnish Diabetes Risk Score (FINDRISC) for diabetes risk assessment. Nevertheless, these systems frequently depend on manually selected, static variables and are not flexible enough to adjust to the changing needs of patient data. By utilizing multimodal data sources, such as genetic information, wearable device data, and electronic health records (EHRs), ML-enhanced models have overcome these constraints and produced more precise and customized risk prediction tools (16, 17) (Figure 2).

#### 2.1.1 Transitioning from traditional scoring systems to ML-enhanced predictive models

The Finnish Diabetes Risk Score (FINDRISC) and other traditional risk-scoring systems have long been used as fundamental instruments for diabetes stratification and prediction. These systems frequently include clinical factors chosen by univariate analysis or expert consensus, and they usually rely on traditional statistical methods that assume linear relationships between variables and outcomes (18). Although these models offer a structured framework for risk assessment, their intrinsic drawbacks, such as their incapacity to capture intricate, nonlinear interactions among predictors, frequently lead to subpar accuracy, especially in patient populations that are heterogeneous (19). Traditional scores are frequently developed on selective cohorts, limiting their generalizability to real-world clinical settings where patient demographics and comorbidities may vary significantly (20).

By addressing these issues through sophisticated computational methods, machine learning (ML) models, on the other hand, offer transformative potential. Neural networks, Random Forest (RF), gradient boosting, and other machine learning algorithms are excellent at spotting complex patterns in high-dimensional datasets, including nonlinear and interactive effects between variables (21, 22). When it comes to predicting diabetes-related outcomes, ensemble techniques like CatBoost have shown better discrimination and calibration than established scores like GRACE or conventional logistic regression (23, 24). These models maximize the informational yield from available datasets by utilizing a wider range of predictors, including clinical, behavioral, and demographic data (25, 26). Notably, ML approaches have been successfully applied to predict diabetic complications and health literacy levels, areas where traditional scores often falter due to oversimplification (27, 28). Although they are useful, traditional risk assessment tools rely on a small number of clinical and demographic variables that frequently lack the dynamic adaptability and granularity needed for personalized medicine (29). In contrast, ML-enhanced models use extensive datasets, such as imaging, clinical, demographic, and even socioeconomic factors, to find intricate patterns and relationships that conventional models might miss (30). ML algorithms like Artificial Neural Networks (ANN), Support Vector Machines (SVM), and K-nearest neighbors (KNN) have shown excellent performance in the context of hip fracture risk prediction, with accuracy levels ranging from 70.26% to 90% and Area Under the Receiver Operating Curve (AUC) values between 0.39 and 0.96 (31). The predictive power of these models is greatly increased by incorporating a variety of variables, such as geometric factors from imaging data and finite element analysis (32).

Beyond traditional risk factors, AI is being used in early screening to identify subtle biomarkers and imaging features that are difficult to detect using traditional techniques. ML models have been created to evaluate multi-target panels from blood tests in colorectal cancer (CRC) screening, with AUC values of up to 0.941 for CRC detection and 0.925 for the classification of colorectal adenoma (CRA) (33).

These models provide a non-invasive, affordable, and scalable approach to early detection, outperforming conventional biomarkers such as CEA and CA199 (34). With an AUC of 0.87 for identifying high-risk prostate cancer (PCa), ML models based on MRI radiomic features have also demonstrated encouraging results in PCa risk stratification, supporting individualized treatment planning. These developments demonstrate how AI can be used to enhance conventional scoring models by increasing their predictive accuracy and integrating a wider variety of data sources.

The capacity of ML-enhanced models to incorporate multimodal data—such as imaging, clinical, and behavioral factors—to produce a thorough risk assessment is one of their main innovations. ML algorithms like Random Forest (RF) and Adaptive Boost Machine (ABM) have been used to predict 90-day functional impairment in stroke risk prediction, with AUC values that are comparable to those of conventional logistic regression models (35). These models provide a more comprehensive evaluation of patient risk by utilizing a wide range of predictors, such as imaging data, clinical history, and lifestyle factors (36). By combining several algorithms to reduce overfitting and increase generalizability, stacking-integrated machine learning (SIML) techniques have further improved these models’ predictive performance (37). With an AUC of 0.877, sensitivity of 81.8%, and specificity of 81.9% in cervical cancer screening, SIML models have greatly outperformed conventional risk assessment instruments (38).

The use of AI in risk assessment and early screening also includes resource allocation and screening interval optimization. AI models like Mirai have been used in breast cancer screening to classify patients into risk groups according to their mammogram data. This allows for customized screening intervals, which lowers the incidence of advanced cancers by about 18 cases per 1,000 screened people (39). By concentrating on high-risk individuals, this strategy not only increases the effectiveness of screening programs but also lessens the strain on healthcare resources (40). Analyzing data from the Short Physical Performance Battery (SPPB), ML models have been developed to predict fall risk. These models have achieved AUC values of 0.78 and offer a scalable solution for identifying older adults who are at high risk of falling (41). These models offer a more precise evaluation of fall risk by combining a variety of predictors, such as handgrip strength, body mass index (BMI), and fall history (42).

Several drawbacks of conventional scoring models, such as their dependence on a small number of predictors and their incapacity to capture intricate relationships between variables, are also addressed by the incorporation of AI and ML in early screening and risk prediction. Conventional stroke risk scores frequently depend on a limited number of clinical predictors, which might not adequately account for the variety of stroke risk factors (43). However, to provide a more thorough evaluation of stroke risk, machine learning models can integrate a variety of predictors, such as imaging data, clinical history, and behavioral factors (44). By continuously adding new data, machine learning models can adjust to shifting risk profiles and gradually increase their predictive accuracy (45).

#### 2.1.2 Multimodal data integration

An innovative strategy for addressing this worldwide health issue is the incorporation of AI into diabetes risk assessment and early screening. AI models can greatly improve the precision and effectiveness of diabetes detection and risk stratification by utilizing multimodal data, such as genetic information, wearable device outputs, and EHRs. The shortcomings of conventional diagnostic techniques, which frequently rely on a single data source and might overlook early signs of disease progression, are addressed by this multimodal approach. AI systems that have been trained on extensive datasets have shown impressive predictive powers. They were able to identify high-risk subgroups for Type 2 Diabetes with an AUC of 0.94 (46). This high accuracy highlights AI's potential to support early detection and preventive measures, which are essential for reducing the serious consequences of diabetes, such as retinopathy, nephropathy, and cardiovascular disease (46).

With its increased risk of T2DM, cardiovascular disease, and poor perinatal outcomes, gestational diabetes mellitus (GDM), a condition marked by glucose intolerance initially identified during pregnancy, poses serious risks to both mother and child (38, 47). Traditional screening techniques, like the oral glucose tolerance test (OGTT), are frequently laborious, time-consuming, and have a limited capacity to predict risk prior to the second trimester, despite the importance of early detection and intervention (43, 47). By combining various data sources, such as clinical, demographic, and multi-omics data, AI-driven models that use ML algorithms present a promising way to accurately and efficiently predict the risk of GDM (41, 48).

While multi-omics techniques offer a thorough understanding of the molecular alterations linked to GDM, ultrasound imaging provides real-time information on fetal and placental development, both of which are essential for early risk assessment (38, 49). The potential of imaging biomarkers in early diagnosis has been highlighted by studies showing that AI models that incorporate placental texture features from ultrasound images can successfully differentiate between pregnant women in good health and those with GDM (38, 49). It has been demonstrated that combining multi-omics data with clinical variables, such as plasma adiponectin levels and other metabolic markers, improves predictive accuracy (47, 50). In addition to improving the accuracy of GDM prediction, these integrated approaches give physicians practical advice for early intervention, such as pharmacological treatments or lifestyle changes, to reduce negative consequences (41, 47).

AI models are especially useful in settings with limited resources because they have shown a remarkable degree of versatility in using readily available clinical variables to predict GDM risk. Traditional risk stratification techniques have been outperformed by non-invasive models that use maternal age, mean arterial blood pressure, prior history of GDM, and ethnicity. These models have demonstrated high predictive performance (AUC: 0.82) (41, 50). These models remove the need for intricate laboratory testing, making them widely applicable in low- and middle-income nations with limited access to cutting-edge diagnostic equipment (41, 43). AI-driven models are useful in a variety of healthcare settings because they have been tuned to achieve high sensitivity and specificity with few input variables (41, 48). A study created 12 distinct machine learning models, four of which, with just 7–12 variables, had a sensitivity of 0.82 and a specificity of 0.72–0.74, indicating the possibility of scalable and effective GDM screening (48).

Beyond risk assessment, the creative application of AI in GDM prediction incorporates long-term health monitoring and tailored interventions. With the use of AI models, patients can be categorized according to their risk profiles, allowing for more focused interventions like medication, exercise regimens, and dietary changes (41). AI has also been used to forecast when GDM will progress to postpartum type 2 diabetes, providing a customized risk score that can direct long-term medical interventions (51). Prenatal fasting glucose levels, gestational age at diagnosis, and insulin therapy during pregnancy have all been found by machine learning algorithms to be significant predictors of postpartum dysglycemia, laying the groundwork for individualized postpartum care (51). These developments demonstrate AI's potential to lessen the long-term burden of diabetes and its associated complications in addition to improving immediate pregnancy outcomes.

Utilizing cutting-edge machine learning methods, like eXtreme Gradient Boosting (XGBoost), to combine various data types is another significant development in this field. When paired with demographic factors, Polygenic Risk Scores (PRS) from genome-wide association studies (GWAS) and Multi-image Risk Scores (MRS) from medical imaging data offer a comprehensive picture of a person's risk for diabetes (46). This integration allows for the identification of subtle patterns and correlations that traditional methods might overlook. The predictive ability of AI models 2 has been further improved by the use of medical imaging methods like electrocardiography (ECG) and abdominal ultrasonography to identify T2D-associated diseases like cardiovascular disease and nonalcoholic fatty liver disease (46). CGM outputs and other wearable device data provide real-time insights into metabolic health, facilitating dynamic risk assessment and individualized intervention strategies (52).

The diversity of diabetes risk factors among various populations is also addressed by the use of AI in diabetes screening. Due to variations in lifestyle, genetics, and environmental factors, traditional risk assessment instruments created for Western populations frequently perform poorly when used with Asian or other ethnic groups (53). However, by training on local datasets, AI models can be customized to target particular populations, increasing their clinical utility and predictive accuracy. When applied to Chinese populations, for example, ensemble machine learning techniques have been demonstrated to perform better than current non-invasive risk score systems, underscoring the significance of developing population-specific models (53). AI is a useful tool for global diabetes prevention initiatives because of its adaptability, especially in areas with inadequate healthcare resources.

AI-driven diabetes screening has the potential to improve patient engagement and adherence to preventive measures, which is another important benefit. Online platforms and other AI-based risk assessment tools offer easily navigable interfaces that enable people to evaluate their risk of diabetes and obtain tailored recommendations (46). With the help of these resources, patients can take the initiative to change their lifestyles, which are essential for preventing diabetes and include better eating and more exercise (52). AI models can help medical professionals spot high-risk patients early, allowing for prompt interventions and lessening the strain on healthcare systems. Clinicians can better monitor patient progress and customize treatment plans by using predictive models that forecast changes in HbA1c levels based on EHR data (52).

These models' clinical applicability is further improved by the application of explainable AI (XAI) techniques, which offer insights into the variables influencing risk predictions. Chest radiographs (CXRs) may be used for improved T2D screening, for example, as XAI has shown correlations between certain adiposity measures and high predictivity (54). In addition to fostering confidence in AI-driven diagnostics, this openness makes it easier to incorporate AI suggestions into clinical procedures. Further demonstrating AI models' scalability and potential for broad use is their capacity to handle massive amounts of data, including more than 270,000 CXRs and 160,000 patient records (54).

Recent advancements show how multi-modal data fusion, which integrates lifestyle, genomic, clinical, and imaging data, can increase prediction accuracy. Nevertheless, there are still challenges in aligning different features while lowering noise and positional shifts (55, 56). By using adaptive region-feature alignment and RoI jittering to address the positional misalignment between modalities, Aligned Area CNN (AR-CNN) increases robustness in object detection tasks (56). Similarly, Frequency-Aware Feature Fusion (FreqFusion) addresses intra-category inconsistency in dense predictions by harmonizing spatial and boundary features using adaptive low- and high-pass filters. This method can be used for imaging analysis associated with diabetes (57). These techniques emphasize the importance of dynamic feature calibration, where spatial and temporal dependencies are modeled to refine feature relevance, as demonstrated by video-based 3D detection systems that propagate scene features across frames (58).

The gap between algorithmic accuracy and clinical interpretability can be reduced by combining statistical models with explainable AI (XAI) frameworks. By tailoring feature selection to specific patient profiles, SHAP value analysis in the iCARE system maximizes diagnostic accuracy for early-stage diabetes prediction by measuring feature contributions (59). Trust-weighted fusion of multi-block features can withstand noise thanks to evidential deep learning (EMFF) and other complementary techniques that quantify uncertainty in adversarial environments. This is an essential capability for diabetes risk stratification using high-dimensional gene expression data (60). Hybrid optimization algorithms demonstrate how statistical optimization and artificial intelligence collaborate to reduce dimensionality by continuously enhancing gene signatures to attain higher classification accuracy (61).

However, for equitable implementation, disparities in data quality between populations must be addressed. The absence of standardized electronic health records (EHRs) in rural and underserved areas exacerbates biases in AI models trained on urban datasets. Large-scale, low-cost retinal imaging is used by solutions like cross-modal self-supervised learning to democratize access, while federated learning frameworks safeguard privacy by decentralizing model training (24, 62). A critical strategy for scalable diabetes screening in resource-constrained settings, ensemble approaches reduce overfitting in unbalanced datasets and achieve 93% accuracy with smaller feature sets (63).

Future directions should prioritize interdisciplinary collaboration to validate fusion techniques across multiple cohorts and standardize multimodal datasets. While integrating AlphaFold-predicted protein structures with EHRs (as in PredGO) may uncover new biomarkers for diabetic nephropathy, real-time edge computing may enable low-latency, contrast-adaptive monitoring of glycemic fluctuations (64, 65). Precision-equitable diabetes care can overcome present constraints by balancing AI's scalability with statistical rigor, providing tailored interventions that are both clinically actionable and widely available.

Explainable AI (XAI) is increasingly recognized as a paradigm-shifting approach in healthcare, particularly for precision-equitable diabetes care. XAI bridges the gap between complex AI-driven predictions and useful clinical insights. By incorporating multimodal data, such as wearable sensor outputs, imaging, and electronic health records (EHRs), XAI enhances the interpretability of federated learning models, which are crucial for collaborative yet privacy-preserving diabetes management across diverse populations (66). Federated learning enables multiple institutions to train a single AI model on various datasets without sharing raw data, addressing disparities in data availability and labeling methods (67). However, clinician trust is often diminished by these models' “black-box” nature. By employing techniques like SHAP (Shapley Additive Explanations) and Local Interpretable Model-Agnostic Explanations (LIME), which elucidate feature importance and decision pathways in glycemic control predictions or diabetic retinopathy screening, XAI tackles this problem (68, 69).

XAI is novel due to its dual capacity to strike a balance between cutting-edge AI and conventional statistical methods like logistic regression or decision trees, which are still essential for risk stratification in diabetes care due to their intrinsic transparency (70). While CNNs and other deep learning models outperform these methods in terms of accuracy, particularly for tasks like retinal image analysis, their opacity makes clinical adoption more challenging (71). XAI addresses this by reducing complicated neural network outputs into human-friendly rules or visualizations. Examples of these include counterfactual explanations that demonstrate how shifting HbA1c levels impact risk predictions or saliency maps that draw attention to microaneurysms in fundus images (72, 73). This synergy is crucial for precision equity because it reduces biases in marginalized populations and ensures that AI tools are both high-performing and accessible to clinicians with varying degrees of technical expertise (71, 74).

One major technological advantage in the treatment of diabetes is XAI's ability to integrate multimodal data fusion techniques, such as REMEDIS, which enhances model robustness by combining representations from multiple data sources. By integrating retinal scans with EHR-derived comorbidities, XAI can demonstrate how diabetic neuropathy detection is enhanced, providing clinicians with a thorough grasp of patient risk (68). XAI's role in federated learning ensures equitable model performance across organizations with diverse data demographics—a critical component of diabetes management globally (67, 74). XAI also makes it easier to comply with legal frameworks like GDPR, which require transparency in automated decision-making, by offering detailed explanations (69, 71).

When clinicians comprehend the model's reasoning, they move from skepticism to trust, which is one of the long-standing problems in diabetes care that XAI can help with (75). Generative counterfactual XAI frameworks can simulate how minor alterations in ECG morphology, a stand-in for diabetic autonomic neuropathy, impact AI predictions in order to conform to clinicians' causal reasoning (73). Similarly, Clinical decision support systems (CDSS) driven by XAI can compare evidence in favor of and against insulin titration, encouraging team decision-making and reducing therapeutic inertia (76, 77). New tools like HistoMapr-Breast demonstrate how XAI can enhance diabetes workflows by pre-identifying diagnostic regions in pathology slides or continuous glucose monitoring trends, despite the fact that they were developed for oncology (68).

XAI is a significant development for precision-equitable diabetes care by demystifying AI outputs, validating federated models across multiple cohorts, and emphasizing the complementary roles of AI and conventional statistics. Future studies should concentrate on improving multimodal data fusion for low-resource settings, standardizing XAI evaluation metrics in clinical settings, and expanding real-world validation of frameworks like LIME-SHAP hybrid models (69, 71). XAI can accelerate the shift from reactive diabetes management to proactive, customized interventions while maintaining equity and trust by placing equal emphasis on interpretability and performance.

Missing values, temporal misalignments, and inconsistent data formats are common problems with multimodal datasets. While digital health records might not include clinical biomarkers, EHRs might not have detailed lifestyle data (78, 79). Robust machine learning models cannot be trained with sparse datasets, so sophisticated imputation methods and FL frameworks are required to reduce bias (80). Scalability is limited by the substantial computational resources required to process high-dimensional data (81, 82). Additionally, privacy concerns arise when integrating sensitive health information across platforms, requiring stringent anonymization protocols (83). Although AI models are very good at identifying patterns, clinical translation is made more difficult by their “black-box” nature. As demonstrated by studies where ML-derived phenotypes were not easily actionable without additional validation, doctors may mistrust predictions that lack mechanistic explanations (79, 84). Efforts to enhance model interpretability—such as attention mechanisms in transformer architectures—are critical for fostering trust (85). External validity is called into question because the majority of multimodal studies concentrate on homogeneous cohorts. In rural areas with different comorbidities and access to healthcare, algorithms trained on urban populations may perform worse (86, 87). Cross-institutional collaborations and diverse dataset curation are essential to address this limitation. Further demonstrating AI models' scalability and potential for broad use is their capacity to handle massive amounts of data, including more than 270,000 CXRs and 160,000 patient records (54).

### 2.2 Complication surveillance & intervention

With previously unheard-of levels of precision, efficiency, and scalability, the application of AI to the monitoring and treatment of diabetes complications represents a revolutionary approach to managing these chronic conditions. Three crucial areas—diabetic retinopathy, diabetic nephropathy, and diabetic foot complications—are especially affected by AI-driven technologies, which use sophisticated computational techniques to improve early detection, prediction, and intervention techniques.

#### 2.2.1 Diabetic retinopathy

A revolutionary development in medical technology is the application of AI to the monitoring and treatment of diabetic complications, especially DR. Early detection and intervention are essential to preventing irreversible vision loss because DR, a major cause of blindness in the working-age population, is frequently asymptomatic (88, 89). In addition to being time-consuming and resource-intensive, traditional diagnostic techniques that depend on ophthalmologists manually analyzing retinal images also have poor patient adherence rates—less than 50% of diabetics follow recommended yearly eye exams (90, 91). However, AI-driven systems provide a scalable, effective, and precise substitute that can address the rising global burden of DR by introducing specialty-level diagnostics into primary care settings (92, 93).

The creation of autonomous AI diagnostic systems, which have shown impressive diagnostic accuracy, is among the most important developments in this field. An AI system for DR detection, for example, exceeded pre-specified superiority endpoints in a pivotal trial with a sensitivity of 87.2% and specificity of 90.7% (94). This FDA-approved system represents a significant advancement in the use of AI in medicine as it is the first of its kind to be implemented in clinical practice (95). The AI system guarantees accurate disease-level output across a range of patient demographics, including differences in age, race, and ethnicity, by delivering real-time clinical decisions at the point of care along with immediate image quality feedback (96, 97). In environments with limited resources, where access to specialized healthcare is frequently restricted, such capabilities are especially important (98).

Novel machine learning architectures, such the Hierarchical Block Attention (HBA) and HBA-U-Net models, have improved AI-driven systems by honing picture segmentation by concentrating on pixel-level details and spatial correlations (99). These models have demonstrated diagnostic accuracies surpassing 99% in identifying vision-threatening DR (VTDR) when paired with hybrid Convolutional Neural Networks (CNNs) and Support Vector Machines (SVMs) (100). The diagnosis of diabetic macular edema (DME), a serious consequence of DR, is made possible by the combination of fundus imaging with optical coherence tomography (OCT), which further improves diagnostic accuracy (101, 102). These developments highlight AI's capacity to identify DR and track its development, enabling prompt interventions that can greatly enhance patient outcomes (103).

Real-world clinical settings have also validated the use of AI in DR screening. A study with a 92% specificity in Western Australian primary care practices showed that using AI systems for DR detection was feasible (104). To improve diagnostic reliability and decrease false positives, AI algorithms must be further refined. However, issues like low disease incidence rates and poor image quality were noted (105). Notwithstanding these obstacles, AI-driven systems' affordability and scalability make them a viable way to close the global DR screening gap, especially in areas with little access to ophthalmologists (106).

Along with its diagnostic potential, AI has demonstrated promise in tracking the progression of DR by analyzing microaneurysm (MA) turnover, a possible biomarker for DR risk. With high sensitivity and specificity, automated tools have been developed to locate MAs, align retinal images from multiple patient encounters, and estimate MA turnover rates (107). Clinicians can optimize patient care by using this capability to evaluate disease activity and customize treatment plans based on real-time data. By enabling patients to have retinal imaging in primary care settings and receive evaluations remotely, AI-driven systems can support telemedicine-based DR screening, removing logistical obstacles and enhancing screening protocol adherence.

It is impossible to overestimate the economic and societal effects of AI-driven DR monitoring and intervention. In addition to being a major cause of blindness, DR places a heavy financial strain on healthcare systems around the globe. For millions of people with diabetes, early detection and treatment of DR can improve their quality of life, lower healthcare costs, and prevent vision loss. AI-driven systems have the potential to completely transform DR management because they can provide high-efficiency, high-accuracy diagnostics at a reduced cost, especially in low- and middle-income nations where the prevalence of diabetes is disproportionately high.

#### 2.2.2 Diabetic nephropathy

A revolutionary development in the treatment of chronic diseases is the incorporation of AI into the monitoring and treatment of diabetic complications, especially diabetic nephropathy (DN). One of the main causes of end-stage renal disease (ESRD) and a serious complication of T2DM, DN is linked to a high rate of morbidity and mortality (108). The intricate relationship between hyperglycemia, hypertension, and dyslipidemia in DN management calls for a multimodal strategy that AI is uniquely suited to provide (109). A I-driven models that use ML and deep learning (DL) techniques have shown great promise in anticipating the course of a disease, improving treatment plans, and facilitating early intervention to reduce complications (110). The ability of AI to forecast disease progression using time-series data and electronic medical records (EMRs) is one of the most promising uses of AI in DN. Recent research has demonstrated that by examining historical data and spotting minute patterns suggestive of early renal decline, AI models can forecast the onset of DN even before clinical symptoms like microalbuminuria manifest (111). When identifying high-risk patients who might benefit from intensive interventions, like statins and antihypertensive drugs, to postpone or stop the progression to ESRD, this predictive capability is especially helpful (112). By examining biomarkers like serum creatinine levels and the urinary albumin-to-creatinine ratio, AI models have been created to forecast the risk of cardiovascular events, which are strongly associated with DN (113). By averting expensive complications and hospital stays, these models not only improve clinical judgment but also lessen the financial burden related to DN (114). The integration of multimodal AI approaches to concurrently model renal, cardiovascular, and metabolic risks represents a transformative paradigm in precision medicine, addressing the interconnected pathophysiology of these conditions (115, 116).

In order to optimize individualized treatment plans for patients with DN, AI is also essential. Through the integration of data from various sources, such as electronic medical records, laboratory results, and patient-reported outcomes, artificial intelligence algorithms are able to determine the most effective treatment plans customized for each patient's unique profile (117). Based on real-time glucose monitoring and predictive analytics, AI-driven decision support systems can suggest changes to dietary regimens, medication dosages, and lifestyle choices (118). AI-powered mobile health (mHealth) apps further improve this individualized approach by giving patients ongoing feedback on their blood sugar levels, medication compliance, and physical activity, enabling them to actively manage their condition (119).

The early detection and monitoring of DN has also been transformed by the use of AI in imaging and diagnostic technologies. AI algorithms have been added to advanced imaging methods, such as femoral intima-media thickness (IMT) measurements and lower extremity arterial ultrasound, to evaluate the burden of peripheral atherosclerosis and forecast the risk of cardiovascular events (38). By giving doctors a more thorough picture of the patient's overall vascular health, these AI-enhanced imaging tools allow for earlier and more focused interventions (39). In order to increase the precision of DN diagnosis and staging and enable more accurate treatment planning, artificial intelligence has also been used in the analysis of renal biopsies and other histopathological data (41). Fundus photography and optical coherence tomography (OCT), which call for specific tools and skilled workers, are the main methods used by AI algorithms for DR detection (97, 120). Studies reveal that portable smartphone-based cameras achieve lower sensitivity compared to tabletop systems, particularly in low-resource settings where image quality is compromised by inadequate lighting or operator skill (121). Additionally, 28.2% of images in real-world screenings had artifacts that prevented AI from grading them, highlighting the necessity of strong preprocessing and quality control procedures (122).

While AI models like the Hierarchical Block Attention U-Net (HBA-U-Net) achieve 99.18% accuracy in controlled datasets, their performance diminishes in multiethnic cohorts or underrepresented populations (123, 124). AI systems trained on homogeneous datasets exhibit higher false-negative rates for darker retinal pigmentation or atypical lesion patterns, exacerbating health disparities (125, 126). The lack of standardized evaluation frameworks further complicates cross-study comparisons and clinical adoption (127).

The use of multimodal AI techniques to simultaneously model renal, cardiovascular, and metabolic risks is a groundbreaking paradigm in precision medicine that tackles the interconnected pathophysiology of these disorders (115, 116). Metabolic disorders such as type 2 diabetes (T2D), cardiovascular diseases (CVD), and chronic kidney disease (CKD) often coexist because they share risk factors such as hypertension, dyslipidemia, and systemic inflammation (128, 129). Traditional unimodal predictive models, which analyze clinical, imaging, or biomarker data independently, have limited prognostic accuracy and clinical utility due to their inability to capture the synergistic interactions between these systems (130, 131). The Kidney Failure Risk Equation (KFRE) ignores cardiovascular comorbidities that exacerbate the course of chronic kidney disease (CKD), despite using biochemical factors to predict renal outcomes (55). CVD risk stratification tools often ignore renal dysfunction, a critical component of cardiovascular mortality (128, 129). Multimodal AI frameworks overcome these limitations by integrating multiple data modalities, including wearable-derived metrics, omics (proteomics, metabolomics), imaging (retinal fundus photography, coronary CT angiography), and electronic health records (EMR), to generate comprehensive risk profiles (130, 132).

Because it can carry out cross-modal representation learning and dynamic feature fusion, multimodal AI is technologically innovative and can uncover latent biological patterns that single-modality models miss. Deep learning architectures like ResNeSt and Transformer-based models have demonstrated remarkable performance when integrating renal ultrasound images, spectral waveforms, and clinical data to diagnose renal artery stenosis (RAS), with accuracies exceeding 80% (133). By detecting microvascular changes indicative of systemic endothelial dysfunction, the combination of non-invasive clinical risk factors and retinal fundus photography improved the prediction of CVD (132). These developments improve feature representativeness even with small multimodal datasets by utilizing semi-supervised autoencoders and transfer learning strategies (134). Notably, multimodal models also address data imbalance problems, which are common in metabolic and renal cohorts, by employing ensemble learning and synthetic minority oversampling (SMOTE) to improve minority-class prediction (134, 135).

The clinical imperative for multimodal AI is highlighted by the need for early risk stratification and customized interventions. T2D patients with concurrent CKD and CVD exhibit distinct proteomic and metabolomic signatures that more accurately predict adverse outcomes than HbA1c alone (134, 136). Multimodal frameworks like MAIGGT, which combine clinical phenotypes and histopathological features, have achieved AUROCs of 0.92 for BRCA1/2 mutation screening, revealing microenvironmental markers linked to metabolic dysregulation (137). These models enable targeted therapies, such as SGLT2 inhibitors, which concurrently lower metabolic, cardiovascular, and renal risks (129, 136). Interpretability tools like SHAP values make modality-specific contributions clear, encouraging collaborative decision-making and fostering clinician trust (132, 137).

The results for patients with DN have been further enhanced by the incorporation of AI into multidisciplinary care models. AI-driven technologies can be used by multidisciplinary teams that include endocrinologists, nephrologists, dietitians, and diabetes educators to expedite care coordination and guarantee that patients receive prompt and efficient interventions (44). In order to lower the risk of complications and increase adherence to care protocols, I-powered platforms can automate follow-up appointment scheduling, monitor patient progress, and notify clinicians of deviations from treatment goals (43). Moreover, it has been demonstrated that AI-enabled patient empowerment devices, like blood pressure monitors, glucometers, and smart tablets, improve glycemic control and self-management practices in high-risk patients (126).

#### 2.2.3 Diabetic foot

The creation of sensor-based remote patient monitoring (RPM) systems is one of the most exciting uses of AI in diabetic foot care. These systems use multimodal sensing technologies, like smart insoles, to continuously monitor temperature, activity levels, and plantar pressure (138, 139). These gadgets can offer patient-facing biofeedback by incorporating AI algorithms, warning users of persistently elevated plantar pressures or temperature imbalances that might be signs of pre-ulcerative conditions. In addition to empowering patients to actively offload, this proactive approach allows medical professionals to take early action, lowering the risk of ulceration and its aftereffects (140, 141). RPM systems support glycemic control, cardiovascular health, and general disease management by being in line with integrative foot care guidelines (142).

AI-driven image recognition technologies are transforming the evaluation and categorization of diabetic foot wounds in addition to sensor-based monitoring. Conventional wound assessment techniques, like the PEDIS index, mainly depend on clinicians' qualitative evaluations, which can be erratic and subjective (143). AI-powered technologies can identify, locate, and measure ulcers with high accuracy (up to 90%) by analyzing wound images using CNNs and deep learning algorithms (144). In addition to improving diagnostic accuracy, these systems help multidisciplinary care teams communicate with one another, which makes treatment planning and resource allocation more efficient. Patients can self-monitor their DFUs at home with AI-based wound monitoring tools, like the smartphone app “MyFootCare,” which offers insightful data on the course of healing and the effects of self-care routines (138). Despite the potential of these tools, issues like usability, data privacy, and the requirement for more extensive validation studies still exist (38).

The creation of customized therapeutic exercise regimens is another cutting-edge use of AI in diabetic foot care. In people with DPN, foot-ankle exercises have been demonstrated to increase muscle strength, range of motion, and gait biomechanics, lowering the risk of ulcers and improving functional outcomes (39, 41). AI-powered platforms, like the Sistema de Orientação ao Pé Diabético (SOPeD), provide personalized workout plans based on each person's physical capabilities and the severity of their condition. These programs, which can be accessed through mobile or web applications, use gamification techniques and real-time feedback to encourage self-management and adherence (43). The potential of these interventions as supplemental treatments in primary and secondary care settings has been highlighted by studies showing that they can result in notable improvements in foot pain, function, and kinematic outcomes (44).

**Figure 3:**
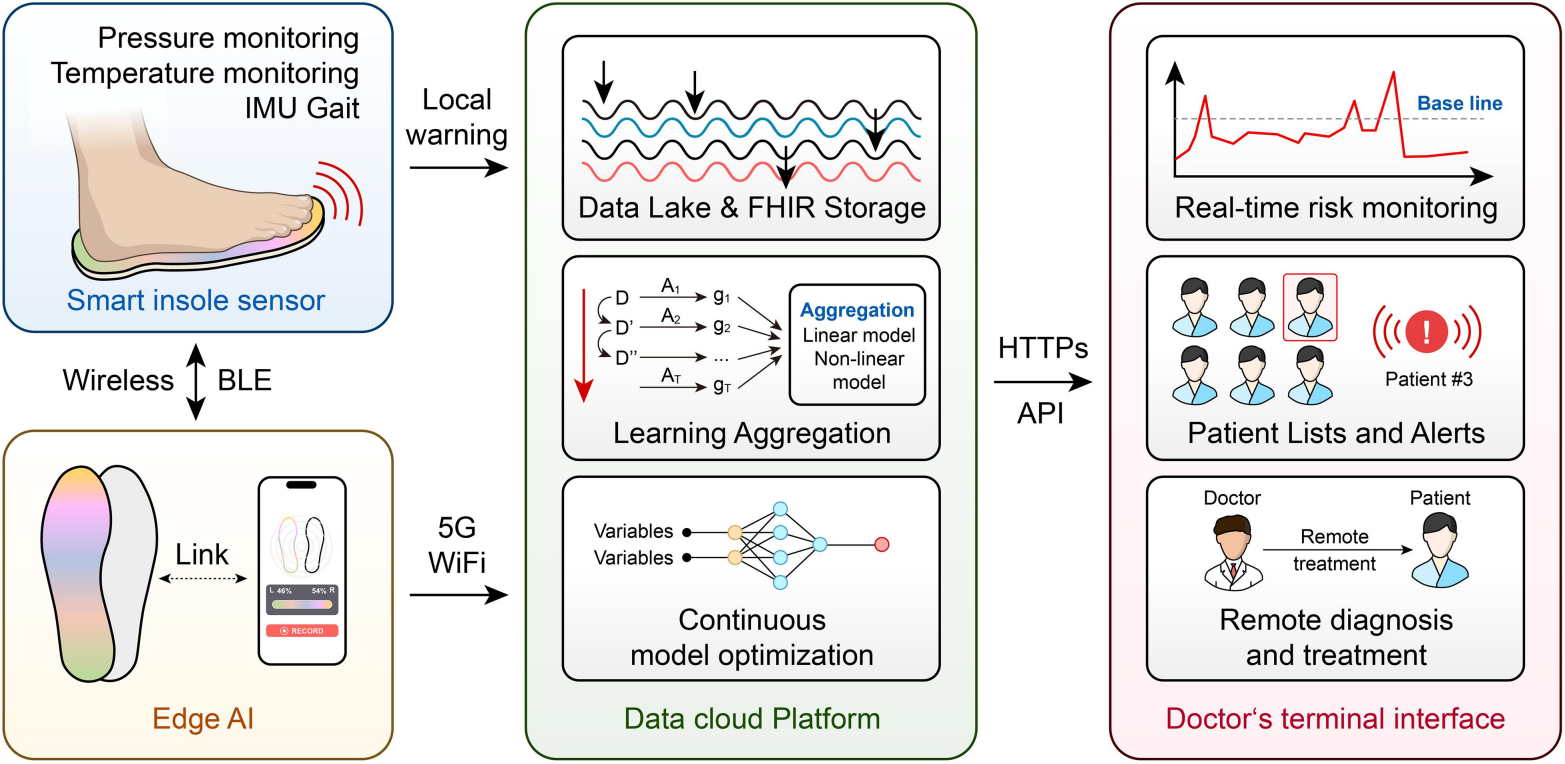
AI-driven closed-loop diabetic foot monitoring.

AI is also being incorporated into decision support and predictive analytics for diabetic foot care. AI models can predict healing trajectories, optimize treatment protocols, and identify risk factors for DFU development by analyzing large datasets (145). In order to categorize people according to their risk of complications and suggest focused interventions, machine learning algorithms can evaluate patient data, including demographic, clinical, and imaging information (146). In addition to improving clinical decision-making, these predictive tools facilitate the application of evidence-based recommendations, like those offered by the International Working Group on the Diabetic Foot (IWGDF) (147). However, issues with data quality, algorithm transparency, and ethical considerations must be resolved for these systems to be deployed successfully (148) (Figure 3).

### 2.3 Real-Time monitoring & decision support

#### 2.3.1 CGM time-series forecasting

**Figure 4:**
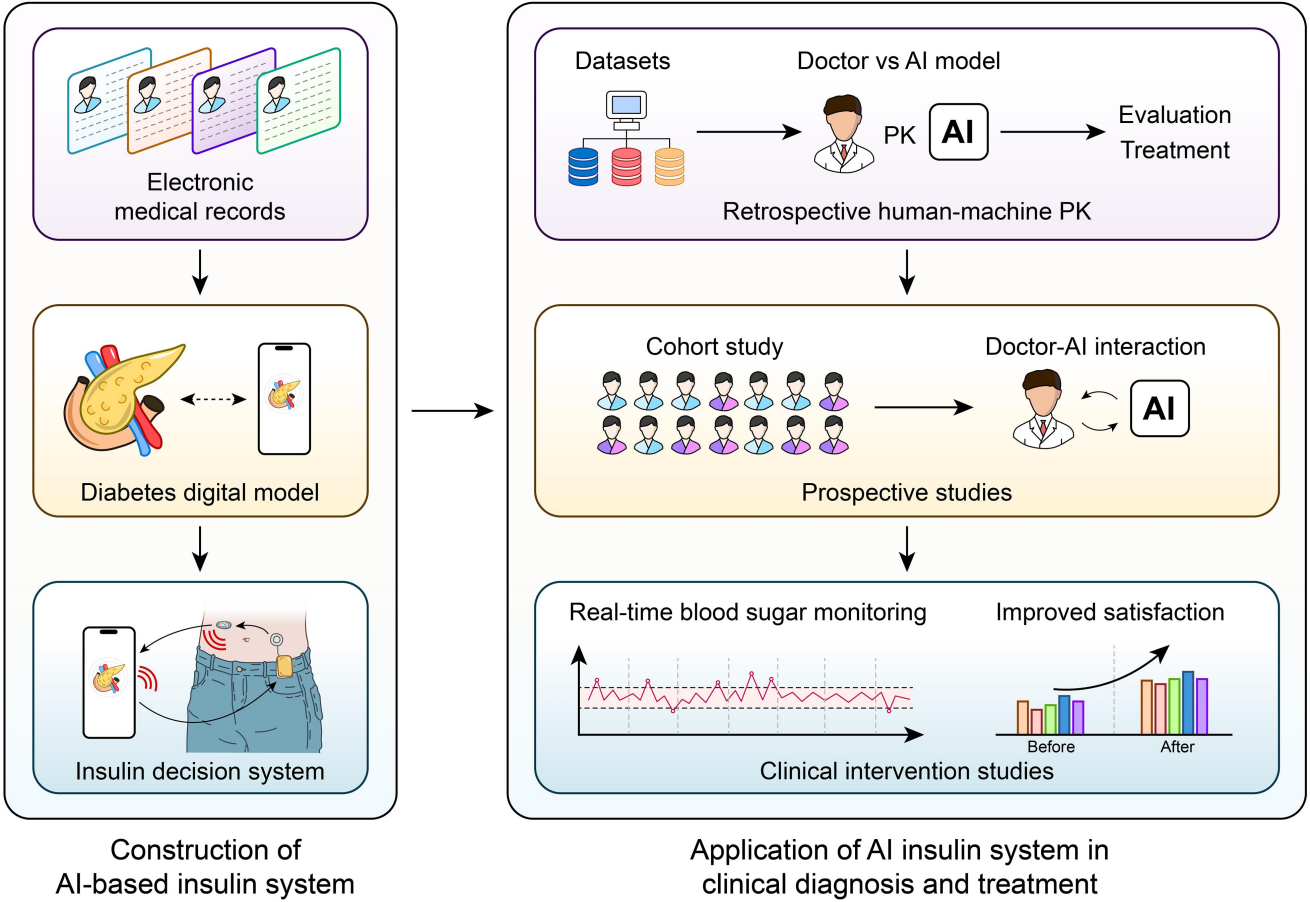
AI-Driven Insulin Titration for T2DM.

A revolutionary method of managing diabetes, especially in the area of time-series glucose prediction, is the combination of AI with wearable technology and CGM devices. In order to optimize insulin dosage and avoid adverse glycemic events, real-time, personalized glucose forecasting is made possible by the combination of AI and CGM data. Because of their exceptional ability to handle sequential data, Transformer architectures and Long Short-Term Memory (LSTM) networks have attracted a lot of attention among the different AI models used for this purpose. LSTM networks—a specialized type of Recurrent Neural Networks (RNNs)—have shown impressive effectiveness in glucose prediction tasks. With a root mean square error (RMSE) reduction from 14.55 to 10.23 mg/dL when compared to standard stacked LSTM approaches in T1D cohorts, research has shown that LSTM models are more successful than traditional machine learning algorithms and even human specialists at predicting blood glucose levels over short time horizons (Shen and Kleinberg, 2025). However, the quality of the data greatly affects this benefit: In real-world use cases where signal integrity is compromised, false hypoglycemia/hyperglycemia alert rates can double due to CGM sensor noise increasing LSTM prediction error by up to 50%. Furthermore, cross-population validation reveals that when applied to older T2D patients with comorbid renal impairment, the majority of LSTM studies rely on data from young adults with T1D. This is explained by their ability to simulate the dynamic and non-linear character of glucose-insulin interactions, which are impacted by elements like insulin administration, food consumption, and physical activity (149). LSTM networks are appropriate for real-world applications where sensor errors and delays are common because they have been demonstrated to be resilient to noise in CGM data (150). A study introduces RL-DITR, a model-based reinforcement-learning framework that optimizes customized insulin dosage for in-patients with type 2 diabetes while also learning patient glucose dynamics (151) (Figure 4). Extensive validation across retrospective, prospective and proof-of-concept trials showed superior accuracy and safety compared with junior/intermediate physicians and near-parity with senior specialists. Seamless integration into the clinical workflow yielded rapid, safe glycemic improvement without increasing hypoglycemia, establishing RL-DITR as a practical, scalable AI decision-support tool for inpatient diabetes care (151).

Conversely, time-series prediction tasks, such as glucose forecasting, are increasingly being investigated using Transformer models, which have transformed natural language processing. Transformers can process long-range dependencies more efficiently than LSTMs because they use self-attention mechanisms to capture relationships between various time steps in the sequence. In glucose prediction, this feature enables better capture of long-cycle patterns, as demonstrated by the CGMformer models (152). In a mixed T1D/T2D cohort (*n* = 427), the LSTM model by Shao et al. was specifically validated for 30-min hypoglycemia prediction with >94% AUC across Chinese and European-American populations, highlighting complementary strengths in short-term accuracy vs. long-range dependency modeling (152). However, Transformers have some significant practical drawbacks in wearable applications: their self-attention mechanism raises dynamic energy by at least the same factor while increasing computational complexity quadratically with sequence length (153). Transformers have demonstrated potential in other fields, but their use in glucose prediction is still in its infancy, and there aren't many studies comparing them to LSTM models. However, early research indicates that Transformers might perform better in situations where complex, multi-scale temporal patterns need to be modeled (154). Transformers may be able to more easily incorporate extra contextual data than LSTMs, such as meal schedules and physical activity, improving prediction accuracy (154).

A number of issues with conventional glucose monitoring techniques are also resolved by integrating AI with CGM systems. The delay between actual plasma glucose levels and interstitial glucose measurements is one of the main problems, which can cause inaccurate real-time predictions. Rate-of-change metrics, variability indices, and other time-based features obtained from CGM data can be incorporated into AI models, especially those using sophisticated feature engineering techniques, to help alleviate this problem (155). Even without additional physiological metrics, these features allow for more precise and timely predictions by capturing the dynamism of glucose fluctuations (155). An important development in this area has been the creation of customized prediction models that are adapted to the glucose dynamics of specific patients. AI models can take into consideration distinct physiological reactions by utilizing patient-specific data, which increases prediction accuracy and dependability (156).

The models' computational efficiency is a crucial component of integrating AI with CGM systems, especially when used on wearable technology. Despite their effectiveness, LSTM networks can be computationally demanding, which presents problems for real-time applications on devices with limited resources. In order to address this problem, efforts to optimize these models—such as by using model compression techniques or lowering the number of parameters—have shown promise (156). Transformers are feasible for wearable applications because they have been modified for edge devices using methods like quantization and pruning, despite their computational complexity (154). One important step toward the development of closed-loop insulin delivery systems, sometimes known as the “artificial pancreas,” is the capability of wearable technology to make precise glucose predictions (119).

Beyond predicting blood sugar levels, AI is also used in CGM systems to detect hypoglycemic and hyperglycemic episodes, which are vital for avoiding serious complications in the treatment of diabetes. Research has shown that AI models, especially deep learning-based models, can accurately classify blood glucose levels into hypo-, normo-, and hyperglycemic ranges (155). While LSTM models perform well over longer prediction horizons, like 1 h, logistic regression models have demonstrated exceptional performance in predicting hypoglycemia within a 15-min horizon (155). These features are crucial for giving patients and medical professionals timely alerts so that proactive measures to preserve glycemic control can be taken (155).

AI and CGM-derived glycemic variability (GV) metrics provide a novel approach to enhancing risk assessment and enabling early, customized interventions in prediabetes—a critical window for preventing the onset of type 2 diabetes (157). CGM technology records dynamic glucose fluctuations and offers granular insights, in contrast to traditional metrics like HbA1c, which often overlook transient dysglycemia in prediabetic individuals (158, 159). AI-driven analytics can be used to systematically evaluate multidimensional GV parameters, such as coefficient of variation (%CV), time in range (TIR), time above/below range (TAR/TBR), and postprandial glucose responses (PPGR), to identify high-risk subgroups (160). Even in well-managed populations, studies show that elevated %CV (>33%) correlates with hypoglycemia risk and suboptimal glycemic control, and post-breakfast glucose excursions are early indicators of dysglycemia progression in prediabetes (161). These metrics are combined with clinical variables like insulin resistance and *β*-cell function by AI models like supervised learning and unsupervised clustering to stratify patients into various “glucotypes” and increase predictive accuracy (158, 162). The integration of CGM-derived glycemic variability (GV) metrics with AI represents a transformative approach to refining risk stratification and enabling early, personalized interventions in prediabetes—a critical window for preventing progression to T2DM (157).

The automated processing of CGM data via AI pipelines addresses the primary shortcomings of manual analysis, enabling scalable, real-time risk assessment. Machine learning algorithms have successfully predicted nocturnal hypoglycemia using state-of-the-art metrics such as the gradient variability coefficient (GVC), which simultaneously measures GV amplitude and hypoglycemia frequency (161). AI-powered meal detection algorithms improve postprandial glucose measurement accuracy by reducing reliance on self-reported logs, which are prone to error (163). These advancements particularly affect marginalized populations, where disparities in diabetes technology access exacerbate health inequalities (162). Notably, AI-enhanced CGM analytics have identified metabolomic signatures associated with GV, suggesting potential biomarkers for early intervention (164). Because longitudinal studies link GV to cardiometabolic outcomes like atherosclerosis and cardiovascular events in prediabetes, its prognostic value goes beyond glycemic control (158, 165).

The combination of CGM and AI may improve personalized prediabetes prevention strategies. Dynamic risk models that include TIR and PPGR could direct tailored lifestyle or medication interventions, such as treating post-breakfast hyperglycemia with dietary modifications (162, 166). Furthermore, there is mounting evidence that short-term GV metrics can be used to forecast long-term complications, offering a useful alternative to HbA1c in resource-constrained settings (159, 167). However, there are still problems, like validating AI models in large-scale trials and standardizing GV thresholds across different populations (160, 168). In order to understand the mechanistic connections between GV and T2DM pathogenesis and eventually enable precision medicine in the management of prediabetes, future research should give priority to integrated multi-omics approaches (158, 164). Clinicians can shift from reactive to proactive care by utilizing AI-driven CGM analytics, reducing the worldwide burden of type 2 diabetes in its early stages.

AI-processed CGV metrics represent a paradigm shift in the management of prediabetes by providing unparalleled granularity in risk assessment and intervention customization. From automated glucotype classification to metabolomic biomarker discovery, these developments bridge the gap between data-rich CGM outputs and useful clinical insights. As technology advances, integrating AI with real-time CGM data will be crucial to achieving early, equitable, and customized diabetes prevention (162, 166).

The integration of AI with CGM systems still faces a number of obstacles, despite the notable progress. The generalizability of AI models across diverse patient populations remains a critical challenge in diabetes management, particularly when addressing the distinct pathophysiological profiles of type 1 diabetes (T1D) and type 2 diabetes (T2D). Long short-term memory (LSTM) networks have shown strong performance in cross-population validation studies, making them a promising tool for hypoglycemia prediction. Their capacity to identify temporal dependencies in continuous glucose monitoring (CGM) data is a significant innovation. For mild hypoglycemia prediction in Chinese cohorts, they achieved area under the curve (AUC) values exceeding 97%, with only a slight 3% decrease when applied to European-American populations (152). This performance outperforms conventional machine learning techniques like random forests (RF) and support vector machines (SVM), which frequently have trouble with false alarm rates—a major obstacle to clinical adoption (152). The LSTM's superiority stems from its capacity to model complex glucose dynamics while maintaining high specificity (reducing false alarms by up to 42% compared to SVM/RF) across both T1D and T2D subgroups (152).

However, resolving the intrinsic constraints in training data diversity is essential to these models' translational potential. When used in global populations or for the management of type 2 diabetes, the majority of early hypoglycemia prediction algorithms may be biased because they were created using small, homogeneous cohorts of Western T1D patients (152). Due to variables that may not be sufficiently represented in training datasets, such as younger age, lower BMI, and higher use of premixed insulin formulations, Southeast Asian T2D patients have distinct risk profiles (152). Although continued validation in underrepresented populations is still crucial, the LSTM's demonstrated generalizability in a validation cohort of 427 patients suggests its adaptability to these heterogeneities (152).

The clinical utility of LSTMs is further improved by technological developments in model interpretability. To verify whether LSTM-based predictions are consistent with physiological principles—a crucial prerequisite for patient safety—tools such as SHapley Additive exPlanations (SHAP) have been used (169). Two LSTM variants with similar accuracy showed significant differences in clinical reliability in one study: only the physiologically consistent model (p-LSTM) correctly learned the inverse relationship between insulin administration and glucose levels, as confirmed by SHAP analysis (169). This emphasizes how crucial it is to incorporate explainability frameworks to guarantee that models generalize across populations both statistically and pathophysiologically (169).

Additional avenues for enhancing generalizability are provided by the combination of ensemble techniques and hybrid architectures. To overcome the drawbacks of single-model approaches, bidirectional LSTM layers combined with transformer encoders, for instance, can simultaneously capture short-term glucose fluctuations and long-term trend (170). Personalized glucose forecasting using such hybrid systems has shown promise, especially when metabolomic or immune response data are included to account for inter-individual variability in T1D progression (170). Similarly, using feature selection algorithms like GALGO, which cut input variables by 50% without compromising accuracy, sex-specific ensemble models for T2D prediction achieved AUCs of 0.96–0.98. This approach could be modified for population-specific tuning (63).

Despite these developments, there are still significant gaps. There is little research on how AI-driven insights translate into customized treatment modifications; instead, the majority of studies concentrate on glycemic prediction rather than therapeutic outcomes (171). Biases in model performance can be sustained by differences in dataset representation, such as the UK Biobank's underrepresentation of female and non-White participants (172). The most predictive factor for the risk of hypertension in T2D patients was sex assigned at birth, although its relative significance differed greatly between datasets with different demographic balances (172). Addressing these disparities requires not only larger, more diverse cohorts but also algorithmic innovations like federated learning to pool data across institutions while preserving privacy (172).

Three areas should be given priority in future directions: multi-omic integration (e.g., metabolomic markers for insulin resistance in T2Dor TCR sequencing for autoimmune risk stratification in T1D) (173). Additionally, real-world hybrid model validation in low-resource environments where CGM adoption is increasing, and standardization of outcome measures to facilitate comparisons between different studies (173). The success of zimislecel, an allogeneic stem cell-derived treatment that restored endogenous insulin production in T1D patients, illustrates the potential for combining AI with biomarker-driven interventions to customize diabetes care (174). Achieving equitable clinical impact will depend critically on AI models' ability to generalize across the whole range of diabetes subtypes, ethnicities, and comorbidities as the field advances toward precision medicine.

Although cross-population generalizability for diabetes management has advanced significantly with LSTM-based models, their ultimate usefulness will depend on thorough validation in practical contexts, improved interpretability frameworks, and proactive mitigation of dataset biases. The combination of wearable technology, immunotherapeutic advancements, and deep learning provides a roadmap for creating truly patient-centric solutions that cut across phenotypic and geographic barriers (175, 176). The models' ability to adjust to abrupt changes in glucose dynamics, such as those brought on by illness or medication changes, is also called into question by their reliance on historical CGM data for training (156).

A paradigm shift in diabetes care is being brought about by the combination of AI with wearable and CGM devices, which presents previously unheard-of possibilities for individualized, real-time glucose prediction. At the forefront of this change are Transformer models and LSTM networks, which have special advantages in managing sequential data. Transformers have the potential to capture intricate, long-range dependencies and may even outperform LSTMs in specific situations, even though LSTM models have proven their effectiveness in short-term glucose prediction. Realizing the full potential of AI in diabetes care will require further development of AI models as well as increases in generalizability and computational efficiency. These technologies have the potential to significantly contribute to the creation of closed-loop insulin delivery systems, which will ultimately enhance the lives of diabetics.

#### 2.3.2 Real-Time decision support

A revolutionary development in the treatment of diabetes is the combination of wearable technology and CGM systems, especially when it comes to real-time decision support for hypoglycemia alerts and meal recommendations. People with diabetes can now make well-informed decisions regarding their insulin dosage, food choices, and physical activity levels thanks to this innovation, which uses the continuous, high-frequency data produced by CGM devices to provide actionable insights. These systems’ real-time functionality fills important gaps in conventional self-monitoring of blood glucose (SMBG) techniques, which frequently offer patchy and delayed data, raising the possibility of hypoglycemic episodes and less-than-ideal glycemic control (4, 177).

The ability to anticipate and prevent hypoglycemia through predictive low glucose alerts is one of the biggest technological benefits of combining CGM with wearables. Predictive low glucose alerts have been shown in studies to dramatically improve patient safety by reducing the frequency and duration of hypoglycemic episodes by up to 59% (178). Because they offer early warnings that enable prompt interventions, like carbohydrate consumption or insulin adjustments, these alerts are especially helpful for people with impaired hypoglycemia awareness or those who are at high risk of experiencing severe hypoglycemia (178, 179). By integrating these alerts with wearable technology, users can minimize disruptions to their routines by receiving notifications in real-time, even while they are involved in daily activities (177).

Apart from preventing hypoglycemia, the incorporation of CGM with wearable technology enables customized meal suggestions, which are essential for maximizing postprandial glucose regulation. When planning food intake, sophisticated algorithms, like those used by the DiaCompanion system, forecast postprandial blood glucose levels using real-time CGM data and offer customized dietary recommendations (180). This method lessens the burden of manual glucose monitoring and dietary planning, which can be especially difficult for people with T1D or GDM. It also helps people avoid hyperglycemic spikes (181). Integrating these suggestions with automated insulin dosing systems has the potential to enable more aggressive insulin adjustments upon meal detection, which can then be confirmed by positive CGM rate-of-change trends. This could lead to fully closed-loop insulin delivery (182).

Numerous studies have validated the practical use of these technologies and shown notable improvements in glycemic outcomes, such as HbA1c level reductions, longer time in range (TIR), and shorter hypoglycemic duration (181). In comparison to intermittently scanned CGM (isCGM), the use of real-time CGM with alert functionality has been demonstrated to increase TIR by 6.85 percentage points, underscoring the additional advantages of real-time data integration and decision support (179). Additionally, combining CGM data with other health indicators like electrodermal activity and heart rate variability improves the precision of hypoglycemia forecasts and offers a more comprehensive approach to diabetes care (183).

The effectiveness of RT-CGM in enhancing glycemic control in pregnant women with T1D and GDM has been shown by randomized controlled trials (RCTs). The CONCEPTT trial demonstrated how CGM can lower maternal hyperglycemia and improve neonatal outcomes, such as a decreased risk of neonatal intensive care unit (NICU) admissions and large-for-gestational-age (LGA) infants (184). Recent studies have explored the integration of AI with RT-CGM, expanding on these findings to provide real-time dietary interventions. One hour after meals, the DiaCompanion I smartphone app forecasts blood glucose levels using AI based on user-provided meal data. Through real-time meal modification, women with GDM can maintain glucose levels within the recommended range, improving glycemic control and reducing the need for frequent clinic visits (180). Because it tackles the dynamic and unique nature of glucose regulation during pregnancy, the use of AI in this context is especially novel. Complex CGM data can be analyzed by AI algorithms to spot trends and forecast glucose excursions, providing personalized dietary advice that takes into consideration variables like insulin sensitivity, physical activity, and meal composition (180). In addition to improving adherence to dietary interventions, this individualized approach gives patients the confidence to actively manage their condition. Additionally, by reducing the need for frequent in-person consultations, the integration of AI with RT-CGM can ease the strain on healthcare systems, which is particularly advantageous in settings with limited resources (185). The capacity of AI-integrated CGM systems to offer real-time feedback and decision support is one of their main technological advantages. AI systems can notify users when hyperglycemia or hypoglycemia is about to occur, enabling prompt actions like insulin administration or dietary changes. During pregnancy, when even brief episodes of hyperglycemia can have serious effects on fetal development, this preventative strategy is especially beneficial (180, 186). Additionally, even when patients are not physically present in the clinic, AI-driven CGM systems can enable remote monitoring, allowing medical professionals to track patients' blood sugar levels in real-time and take appropriate action (187). Additionally, there is potential for increasing the precision and dependability of glucose monitoring through the integration of AI with CGM. It has been demonstrated that the Dexcom G6 CGM system gives pregnant women accurate glucose readings across a number of sensor wear sites, with a mean absolute relative difference (MARD) of 10.3% when compared to reference values (184). These systems can further improve accuracy by correcting for sensor errors and supplying more trustworthy data for clinical decision-making when paired with AI algorithms. This is especially crucial during pregnancy, when careful glucose management is necessary to reduce the chance of negative consequences (184). The broad use of AI-integrated CGM systems still faces obstacles in spite of these developments. These include creating user-friendly interfaces, integrating these technologies into current healthcare workflows, and requiring strong clinical validation. More research is required to assess the long-term advantages and cost-effectiveness of these systems, especially in healthcare settings with a variety of patient populations (180). An important advancement in the control of blood sugar levels during pregnancy is the combination of AI with wearable and continuous glucose monitoring devices. These systems have the potential to enhance glycemic control, lower the risk of negative outcomes, and enable patients to actively manage their condition by offering real-time, customized dietary interventions.

#### 2.3.3 Telemedicine

In environments with limited resources, like India and Africa, the combination of wearable and CGM technologies with telemedicine has become a game-changer for managing diabetes. Because of the severe infrastructure, cost, and accessibility issues with healthcare in these areas, cutting-edge approaches like telemedicine and CGM are essential to enhancing patient outcomes. In order to close gaps in care delivery, mobile health (mHealth) technologies have become essential in India due to the sharp increase in non-communicable diseases (NCDs), especially diabetes. Utilizing India's vast mobile communications network, mobile health platforms have demonstrated promise in risk assessment, early diabetes detection, and establishing connections between primary care doctors and community providers (188, 189). The viability and acceptance of such approaches in rural and remote areas are demonstrated by initiatives such as the Jodhpur School of Public Health's community-based strategy, which uses mobile health technology to survey households and triage people at high risk for diabetes (4, 189). HealthCubed provides a comprehensive solution for point-of-care testing with its integrated devices, which measure various physiological and biochemical parameters related to cardiovascular diseases. With cloud-based data storage and Bluetooth control, these gadgets are especially useful in rural and semi-urban areas with limited access to diagnostic labs (190, 191). By incorporating these technologies into telemedicine frameworks, diabetes management in marginalized populations is improved through real-time monitoring, tailored feedback, and prompt interventions.

The use of telemedicine and CGM has demonstrated promise in filling important gaps in diabetes care in Africa, where healthcare systems are frequently overburdened by conflicting health priorities. In low-resource settings, continuous glucose monitoring—which has historically been limited to high-income nations due to cost—could be extremely important, especially for monitoring serious conditions like pediatric hypoglycemia in hospitalized children (192). A study conducted in Malawi examined the appropriateness and acceptability of CGM among individuals with T1D at first-level hospitals, emphasizing its potential as a part of individualized diabetes care in settings with limited resources (193). Although the necessity of health education sessions to support its adoption was underlined, participants and healthcare providers reported that the use of CGM was appropriate and acceptable (193). By facilitating remote monitoring and real-time data sharing with healthcare providers, the integration of CGM with telemedicine platforms may further increase its usefulness and remove geographical barriers to care. To guarantee widespread adoption, however, issues like high temperatures, humidity, and restricted access to dependable electricity that can impair CGM sensor performance must be resolved (193).

Real-time data collection, remote monitoring, and customized interventions are just a few of the technological benefits that come with the combination of CGM and telemedicine. For people with type 2 diabetes, the Onduo Virtual Diabetes Clinic (VDC) offers comprehensive care through a mobile app, connected devices, remote lifestyle coaching, and live video consultations with endocrinologists. The viability of remotely prescribing and delivering CGM devices without in-office training was highlighted by the VDC model's notable improvements in HbA1c levels and patient satisfaction (181, 194). By incorporating smartphone apps and wearable fitness trackers into diabetes self-management education and support (DSMES) programs, behavioral goal monitoring has been made easier, allowing teachers to more efficiently monitor patients' adherence to dietary and exercise regimens (188, 195). These innovations highlight the potential of connected health interfaces to enhance patient engagement and improve clinical outcomes.

## 3 Representative models and performance comparison

### 3.1 AI comparison and assessment of model performance, interpretability, and external validity

With notable improvements in accuracy, efficiency, and scalability, the use of AI in risk prediction and early diabetes screening has become a game-changing strategy in healthcare. When it comes to diabetes risk prediction, undiagnosed case identification, and high-risk population stratification, AI-based models—especially those that use ML algorithms—have outperformed conventional statistical techniques (53, 196). These models produce predictive insights without the need for expensive laboratory testing by utilizing a variety of readily available and non-invasive clinical features, including age, gender, BMI, family history of diabetes, and lifestyle factors (51, 197). When it comes to detecting undiagnosed T2D, ensemble approaches that combine several risk score systems have demonstrated improved predictive accuracy, surpassing individual risk score systems (53). The robustness of gradient boosting machines (GBM) and random forest (RF) algorithms in diabetes risk prediction has been demonstrated by their high AUC values (198, 199). AI is now a feasible tool for widespread diabetes screening since its incorporation into primary care and community settings has further decreased detection costs and increased cost-effectiveness (200).

The capacity of AI models to capture intricate, non-linear relationships between risk factors—which conventional statistical approaches frequently oversimplify—is one of the major advancements in this field (51, 198). Novel risk factors that were not previously taken into account by traditional risk prediction models, such as a preference for sweet flavors and urine glucose levels, have been discovered by machine learning algorithms (198). More precise and nuanced risk stratification is made possible by this capability, especially in diverse populations with a range of genetic, environmental, and lifestyle traits (53, 196). The distinct epidemiological characteristics of particular populations, like rural Chinese cohorts, where conventional Western-based risk scores perform poorly, have been addressed by AI models (53, 198). These models' increased predictive accuracy through the use of population-specific data emphasizes the significance of regional risk assessment instruments (53, 196).

Numerous datasets and environments have been used to thoroughly validate the effectiveness of AI-based diabetes prediction models. Using data from the Korean National Health and Nutrition Examination Survey (KNHANES), studies comparing ML-based models with conventional statistical methods discovered that ML models consistently produced higher AUC values, ranging from 0.788 to 0.819, than their statistical counterparts (196). Strong predictive performance was shown by models created with Henan Rural Cohort Study data; depending on whether laboratory data was included, AUC values ranged from 0.767 to 0.872 (198). These results highlight AI's potential to improve early diabetes detection and enable prompt interventions, especially in settings with limited resources and restricted access to cutting-edge diagnostic tools (197, 200).

Through methods like SHapley Additive exPlanations (SHAP) and decision tree analyses, AI models provide interpretability and transparency in addition to their predictive power. These techniques help clinicians make well-informed decisions and customize interventions for particular patient profiles by shedding light on the relative significance of individual risk factors (201). The most important predictors of diabetes, according to decision tree analyses, are age, family history of the disease, and BMI. These findings inform focused community interventions and public health initiatives (197). Proactive diabetes management and prevention have been made possible by the combination of AI with wearable technology and EHR, which has allowed for real-time monitoring and customized risk assessment (38, 52).

### 3.2 Comparison of methodological limitations

A revolutionary but challenging area of healthcare innovation is the combination of AI-based models and traditional statistical methods in precision-equitable diabetes care. While AI-driven methods like deep learning (DL) and machine learning (ML) have proven remarkably effective in tasks like diabetic retinopathy (DR) screening—achieving sensitivities and specificities exceeding 85%—their reliance on large-scale, high-quality datasets creates issues with data standardization, privacy, and equitable access (128). In settings with limited resources, disparities in data annotation quality across regions and the computational requirements of convolutional neural networks (CNNs) may make inequality worse (8). On the other hand, traditional statistical techniques like logistic regression or LASSO provide interpretability and transparency, which are essential for clinical trust. However, they frequently fail to handle the nonlinear, high-dimensional interactions present in diabetes complications like diabetic nephropathy (DN) or cardiovascular disease (CVD) (202). The hybrid use of XAI frameworks, such as XAI4Diabetes, attempts to bridge this gap by combining the predictive power of algorithms like XGBoost (which achieved perfect accuracy in DM classification) with interpretable decision pathways, enabling clinicians to validate model outputs and address biases (203). While traditional methods run the risk of oversimplifying intricate metabolic interactions, AI models trained on heterogeneous datasets may unintentionally perpetuate disparities if they are not calibrated for demographic variability (24, 204). Innovations like federated learning and synthetic data generation could mitigate these limitations by enabling collaborative model training without centralized data sharing, thus preserving privacy and enhancing generalizability (205). Furthermore, the ethical imperative of equitable care demands rigorous validation of AI tools across diverse populations, as seen in studies highlighting the variable performance of DR screening algorithms (206). Ultimately, the synergy of AI and statistical methods must prioritize not only precision but also inclusivity, ensuring that advancements in diabetes care—from early detection of diabetic foot ulcers (DFU) to personalized risk stratification for diabetic ketoacidosis (DKA)—are accessible across socioeconomic and geographic divides (207). Future research should focus on harmonizing algorithmic transparency with clinical utility, leveraging multimodal data integration, and addressing the “last-mile” challenges of implementation in low-resource settings (208).

### 3.3 Comparison of subgroup applicability for different populations

The applicability of AI-based models vs. conventional statistical techniques in diabetes care across diverse population groups represents a critical area of investigation, given the growing global burden of diabetes and the need for personalized, scalable solutions. AI-driven approaches, including machine learning (ML) and deep learning (DL), have demonstrated superior performance in diabetes classification, risk stratification, and complication prediction compared to traditional methods like logistic regression or Chi-square tests (204). XGBoost achieved perfect accuracy (1.0), sensitivity, and specificity in classifying diabetes using sociodemographic and clinical data, outperforming conventional statistical models such as Student's *t*-test (204). However, the generalizability of these AI models across heterogeneous populations—particularly underrepresented groups—remains a challenge due to biases in training datasets, which often overrepresent Western cohorts (62). This disparity is exacerbated in low-resource settings, where limited data availability and infrastructural constraints hinder the deployment of AI tools, despite their potential to mitigate healthcare inequities (202).

Conventional statistical techniques, while less computationally intensive, offer advantages in explainability and interpretability, which are crucial for clinical adoption. Fuzzy cognitive maps (FCMs), for example, enable scenario-based simulations to elucidate variable relationships in diabetes risk prediction, a feature often lacking in “black-box” AI models (204). Such transparency is vital for addressing ethical concerns, including algorithmic bias and data privacy, which disproportionately affect marginalized populations (209). In contrast, AI's strength lies in its ability to process high-dimensional data and identify non-linear patterns imperceptible to traditional methods (209). AI algorithms analyzing breath samples for hypoglycemia detection achieved non-invasive monitoring, a breakthrough unattainable with conventional statistics (209). Yet, these innovations must be validated across diverse demographics; studies note that AI performance degrades in subgroups with differing disease prevalence (210).

The integration of AI with telehealth and wearables further underscores its transformative potential for population-specific care. Mobile apps leveraging AI to tailor dietary recommendations for diabetic patients have shown promise in improving glycemic control, particularly in urban, tech-literate cohorts. However, rural or elderly populations may face barriers due to digital literacy gaps or limited access to wearable devices (202). Conversely, conventional methods like risk scores remain widely applicable in low-tech settings but lack the dynamic adaptability of AI to individual behavioral or physiological changes (204).

Ethical and practical challenges persist in both paradigms. AI requires large, annotated datasets for training, raising concerns about data privacy and consent, especially in vulnerable groups (62). Retrospective studies, though scalable, often fail to capture real-world diversity, while prospective trials are constrained by sample size (211). Conventional techniques, though simpler, struggle with complex interactions (212). Future research must prioritize inclusive dataset curation, hybrid models combining AI's predictive power with statistical explainability, and rigorous subgroup analyses to ensure equitable applicability. Policymakers should incentivize collaborations between AI developers and local healthcare providers to address context-specific barriers, from algorithmic bias to resource limitations (8, 202).

While AI-based models excel in precision and scalability for diabetes care, their applicability varies significantly across populations due to data biases and infrastructural disparities. Conventional statistical methods remain indispensable for interpretability and broad accessibility but are limited in handling complex, real-time data. A synergistic approach—leveraging AI's analytical prowess where feasible and statistical robustness where necessary—could optimize diabetes management globally, provided it is grounded in ethical frameworks and tailored to local needs (202).

### 3.4 Comparison of model updating and iteration capability

The integration of artificial intelligence (AI) and machine learning (ML) models into diabetes care has introduced transformative capabilities in predictive accuracy, personalized intervention, and dynamic model updating, surpassing the limitations of conventional statistical techniques. Unlike traditional methods such as logistic regression, which rely on static assumptions and linear relationships, AI/ML models excel in capturing nonlinear interactions and adapting to evolving clinical data, thereby enhancing their prognostic and diagnostic utility. Random forest (RF) and k-nearest neighbors (KNN) algorithms have demonstrated superior performance in predicting cardiometabolic risk and HbA1c control, outperforming conventional approaches by accounting for complex feature interactions and temporal data shifts. This adaptability is particularly critical in diabetes management, where patient-specific factors and evolving clinical guidelines necessitate continuous model refinement to maintain accuracy.

A key innovation of AI-based models lies in their iterative updating mechanisms, which address the “calibration drift” inherent in dynamic healthcare environments. Conventional statistical models often degrade over time due to shifts in population demographics, clinical practices, or biomarker distributions 26. In contrast, AI/ML frameworks employ proactive strategies such as real-time recalibration, incremental learning, and batch updates to mitigate performance decay. Reinforcement learning (RL) has been leveraged to optimize anesthetic dosing in diabetes-related surgeries, dynamically adjusting to patient responses and operational stimuli (213). Similarly, ensemble methods like gradient-boosted trees and long short-term memory (LSTM) networks enable longitudinal prediction of diabetes progression by integrating time-series data from electronic health records (EHRs) and wearable devices (214). These capabilities are further enhanced by IoT-enabled real-time monitoring, which provides continuous data streams for model retraining—a feature absent in conventional techniques (203).

The transparency and interpretability of AI models also contribute to their clinical adoption. Feature importance analysis in RF and neural networks elucidates the impact of variables such as exercise habits, dietary patterns, and medication timing on glycemic control, facilitating shared decision-making between clinicians and patients (52). This contrasts with the “black-box” perception of early AI systems and aligns with the need for accountability in healthcare. However, challenges persist, including the requirement for large, diverse training datasets to ensure generalizability and the ethical considerations of data privacy (215). Policy initiatives must therefore prioritize standardized protocols for model validation, interoperability, and practitioner training to bridge the gap between algorithmic potential and real-world implementation (216).

AI/ML models represent a paradigm shift in diabetes care by combining predictive precision with adaptive iteration, outperforming conventional statistical methods in scalability and responsiveness. Prospective studies to confirm long-term efficacy across diverse populations and hybrid approaches that combine domain-specific knowledge with AI-driven insights should be the main focus of future research (214). By addressing these frontiers, AI can solidify its role as a cornerstone of precision medicine in diabetes management.

### 3.5 Technical differences and performance trade-offs of diabetes-related models

The creation and use of various predictive and decision-support models, each with unique methodological advantages and disadvantages, has greatly influenced the field of diabetes care. Machine learning (ML)-based predictive systems and hybrid care pathways are two examples of how knowledge-driven and data-driven approaches diverge critically. Knowledge-driven models, like the Clinical Decision Support (CDS) system, incorporate static clinical guidelines and use established risk equations from frameworks like the UKPDS-OM2 to simulate long-term outcomes and cost-effectiveness (217). In contrast, data-driven ML models, including XGBoost and Random Forest, prioritize feature engineering and ensemble learning to optimize predictive accuracy for diabetes-related complications, achieving AUCs >0.75 through Monte Carlo Cross-Validation (218). The hybrid care pathway model, which integrates both paradigms, demonstrates a 13.12% probability of cost-effectiveness at a €45,000/QALY threshold, though its performance hinges on variables like HbA1c reduction and complication costs (219).

Technological disparities further underscore model divergences. While the CDS system relies on deterministic simulations in Excel® to project complications and mortality, ML models dynamically adapt to EHR-derived features, such as age-diagnostic code pairs, to identify high-impact predictors for complications like amputations (218). Notably, ML models exhibit superior robustness in handling imbalanced datasets through stacking architectures and SHAP-based interpretability, enabling granular feature impact analysis (220). However, knowledge-driven models maintain an edge in clinical translatability by aligning with ADA guidelines, whereas ML models face challenges in generalizability due to sample size limitations and “human bias” in real-world prescription patterns (221).

Trade-offs between scalability and precision are revealed by performance comparisons. Better short-term glycemic control (OR 1.73) and lower long-term complication risks were associated with the hybrid model of the SingHealth Diabetes Registry's 43%–59% concordance with doctor prescriptions for antiglycemic and lipid-lowering medications (221). Conversely, although their retrospective design restricts real-time applicability, unsupervised anomaly detection models stratify high-utilization T2DM patients by identifying outliers with 7.94 annual hospitalizations (compared to 3.12 in typical cohorts). An additional level of complexity is introduced by managed care evaluations: When compared to Fee-for-Service enrollees, Medicare Advantage (MA) beneficiaries show higher rates of preventive care but worse intermediate outcomes (such as elevated HbA1c) and lower adoption of GLP-1RAs/SGLT2is, underscoring systemic disparities in evidence-based therapy access (222).

This is demonstrated by the SynthA1c encoder model, which combines clinical data and image-derived phenotypes (IDPs) to predict diabetes risk with an 87.6% sensitivity—a paradigm shift from conventional lab-based classifiers (223). Similarly, feature selection (FS) techniques in ML models reduce dimensionality without sacrificing accuracy, peaking at 150 features for amputation prediction (218). Future directions must address scalability, equity, and hybrid model optimization through prospective validation (222). Ultimately, the convergence of interpretable ML and guideline-driven logic promises to redefine personalized diabetes care, balancing predictive power with clinical feasibility.

The divergence between representative models reflects a broader tension between clinical tradition and computational innovation. While knowledge-driven systems anchor decisions in established guidelines, data-driven approaches uncover latent patterns in heterogeneous datasets. Their comparative performance underscores the need for context-aware deployment—whether prioritizing cost-effectiveness, precision, or equity (218). In order to ensure that models like SynthA1c or CDS can easily integrate into changing care frameworks while addressing biases in data and delivery, future research must prioritize interoperability (223).

### 3.6 Conditional effectiveness of diabetes care models

The effectiveness of diabetes care models hinges on their ability to address patient heterogeneity, systemic barriers, and contextual factors, which often determine their success or failure. Shared Medical Appointments (SMAs), for instance, demonstrate success in improving glycemic control and self-management among diverse populations, particularly when they incorporate culturally tailored interventions and address social determinants of health (SDOH) (224). For African American populations, SMAs that integrate family support, community networks, and culturally sensitive education show higher engagement and satisfaction, bridging gaps left by traditional care models (224). However, their implementation often falters in practices lacking key implementation champions, stable staffing, or additional resources—conditions identified as necessary for SMA success through Qualitative Comparative Analysis (QCA) (225). Without these enablers, logistical challenges such as workflow disruptions and patient recruitment barriers undermine scalability (225).

Precision Medicine (PM) and Simulation Modeling offer another lens into conditional success. Dynamic simulation models excel in evaluating PM interventions by capturing patient-specific pathways and heterogeneity, which static models like Markov chains cannot (226). These models succeed when applied to complex decision cascades, such as personalized treatment selection in type 2 diabetes (T2DM), where they outperform traditional cost-effectiveness analyses by integrating real-world variability (227). However, their failure modes emerge in settings with insufficient data granularity or when assumptions about rational decision thresholds (e.g., in Decision Curve Analysis) misalign with clinician behavior (227). While PM models can optimize drug choices for relapsing-remitting multiple sclerosis, their clinical utility diminishes if patient preferences or safety profiles are inaccurately weighted (227).

Agent-Based Models (ABMs) further highlight the role of context in diabetes care innovation. ABMs simulate social determinants like dietary habits and peer influence, proving valuable for obesity-related T2DM prevention (228). Their strength lies in modeling emergent behaviors, but they often fail to translate into policy due to lack of stakeholder collaboration or oversimplified representations of socioeconomic barriers (228). The absence of ABMs in real-world policy implementation underscores the gap between theoretical potential and practical adoption (228).

Machine Learning (ML) and Predictive Modeling reveal similar conditional dynamics. ML models identify multifactorial predictors of Diabetes Self-Management Education (DSME) engagement, such as socioeconomic status and comorbidities, enabling targeted interventions (229). Yet, their success is limited by data biases (e.g., underrepresentation of minority groups) and algorithmic opacity, which may perpetuate disparities if not addressed (229). While ML can stratify Medicare beneficiaries by DSME participation risk, its recommendations may overlook cultural preferences for triadic decision-making (patient-family-clinician) observed in Black communities (230).

Integrated Care Models, such as the Chronic Care Model (CCM), succeed when they emphasize patient-centeredness and community support, as seen in high patient satisfaction scores for family-involved care (231). However, they fail in fragmented health systems where provider coordination is weak or where SDOH (e.g., transportation, income) are unaddressed (232). The legacy effect of early glycemic control in pediatric diabetes further illustrates this: models ignoring metabolic memory or socioeconomic drivers underestimate long-term complications, leading to flawed economic evaluations (233).

The performance of diabetes care models is deeply contextual. SMAs thrive with champions and resources; PM and simulation models require robust data and alignment with clinical realities; ABMs need stakeholder co-production; and ML must mitigate biases. Future efforts should prioritize adaptive implementation frameworks—like PRISM or QCA—to tailor models to local conditions (225), while advancing equity-focused validation in underrepresented groups (224). Only by addressing these conditional determinants can innovations transition from theoretical promise to real-world impact.

## 4 Unresolved challenges

The potential of AI applications in diabetes care is often limited by disparities in data formats, coding standards, and privacy concerns, which frequently impede smooth data exchange and interoperability (234).

The heterogeneity of health data, which includes multimodal data from wearables, unstructured PGHD, and structured EHRs, is one of the main obstacles. Although AI tools like ML algorithms have demonstrated promise in examining these datasets to identify connections between genetic predispositions, lifestyle factors, and disease outcomes, a major obstacle is the absence of standardized terminology and interoperability frameworks (235). The accuracy of AI models may be jeopardized by problems with data redundancy, incompleteness, and time validation that must be resolved in order to integrate PGHD into EHRs (234). In addition, the lack of widely recognized guidelines for data interchange and representation, like Fast Healthcare Interoperability Resources (FHIR), makes incorporating AI solutions into current healthcare processes even more challenging (236).

Concerns about security and privacy pose a significant obstacle to the use of AI in diabetes treatment. Because health data is sensitive, strict measures must be taken to protect patient confidentiality. However, integrating AI frequently requires using third-party platforms and cloud-based architectures, which may not always adhere to legal frameworks (235). This undermines confidence in AI-driven healthcare solutions by creating vulnerabilities that could expose patient data to cyber threats or unauthorized access. To guarantee fair and efficient treatment for every patient, the ethical ramifications of AI, such as algorithmic bias and the possibility of an excessive dependence on automated systems, must also be addressed (8).

Addressing the issues of data standardization and interoperability requires coordinated efforts in order to fully realize AI's potential in diabetes care. This entails creating standardized data protocols, using open-source data processing platforms like Hadoop and Spark, and putting strong privacy-preserving measures in place (235). Establishing frameworks that enable the safe and effective integration of AI into clinical practice requires cooperation between stakeholders, including legislators, technology developers, and healthcare providers. More research is required to verify how well AI models function in practical situations and investigate new uses, like AI-powered decision support systems and the results of robotic surgery in diabetic patients (209).

Although the use of AI in diabetes treatment has the potential to revolutionize the field, there are many obstacles to overcome, especially with regard to algorithmic bias and ethical governance. AI tools, such as machine learning and deep learning, have shown impressive potential in improving patient outcomes, treatment strategy optimization, and diagnostic accuracy in the management of diabetes (7, 8). AI-driven solutions have demonstrated promise in creating individualized treatment plans and automating ongoing glucose monitoring, enabling patients to make knowledgeable decisions about their care (237). These systems' effectiveness, however, depends on the caliber and representativeness of the training data, which frequently introduces biases that can disproportionately impact marginalized communities (238, 239).

Incomplete or skewed datasets, a lack of diversity in the development teams, and the intrinsic limitations of the algorithms themselves are some of the causes of algorithmic bias in AI systems (238, 239). With sensitivities ranging from 50.98% to 85.90%, a multicenter validation study of AI-based DR screening systems showed notable performance disparities, underscoring the variation in algorithmic efficacy across various population (8). Biases like these can result in unfair healthcare outcomes, especially for underrepresented groups that may already have trouble getting high-quality care (239). Furthermore, a strong ethical framework is required for the incorporation of AI into clinical workflows in order to address issues with patient autonomy, transparency, and data protection (237, 240).

Fairness, accountability, and openness must be given top priority in ethical governance for AI technologies used in diabetes care. Although they offer a fundamental framework, current legal frameworks frequently overlook the complex ethical conundrums raised by AI technologies (238). AI algorithms may perform inconsistently in various healthcare settings due to a lack of standardized evaluation procedures, which could erode public confidence in these systems (241). AI-generated recommendations’ ethical ramifications also need to be carefully examined, especially in light of how they may affect patient autonomy and the doctor-patient relationship (242). Healthcare professionals must make sure that AI-based interventions support patient-centered care rather than obstruct it, and patients must maintain the freedom to accept or reject them (242).

A multifaceted strategy that takes into account technical, ethical, and regulatory aspects is needed to lessen these difficulties. To guarantee consistent performance across various populations, technical efforts should be focused on enhancing data quality and representativeness, creating algorithms that are resistant to biases, and putting strict validation procedures in place (8, 238). Developing inclusive design principles that put the needs of underserved communities first and establishing precise rules for the ethical application of AI in healthcare are two examples of ethical considerations (239, 242). The special difficulties presented by AI technologies, such as the requirement for third-party assessments, openness in algorithmic decision-making, and systems for ongoing observation and enhancement, require regulatory frameworks to change (238, 241).

A paradigm shift in healthcare delivery is also required for the integration of AI into diabetes care, with an emphasis on patient empowerment and interdisciplinary collaboration. In order to create AI solutions that are not only technically sound but also morally and socially responsible, healthcare providers, technologists, and legislators must collaborate (238). It is imperative that patients actively participate in the creation and application of these technologies, guaranteeing that their preferences and values be upheld (242). In order to give patients and healthcare professionals the information and abilities they need to successfully navigate this quickly changing environment, educational programs should be put in place to increase awareness of the possible advantages and hazards of AI in diabetes care (238).

While AI-driven tools, such as automated insulin delivery (AID) systems and clinical decision support systems (AI-CDSS), have demonstrated efficacy in glycemic control and risk stratification (7, 243), their equitable deployment remains hindered by data standardization issues, algorithmic bias, and heterogeneous clinical validation. AI-based DR screening systems exhibit wide variability in sensitivity (50.98%–85.90%) and negative predictive values (82.72%–93.69%) across multicenter studies, raising concerns about generalizability to diverse populations (244). Similarly, the reliance on large, high-quality datasets for training AI models exacerbates disparities in low-resource settings, where data collection infrastructure is often inadequate (245).

A critical unresolved controversy lies in the trade-off between algorithmic performance and interpretability. Deep learning models, though highly accurate, often function as “black boxes,” limiting clinician trust and adherence to AI-generated recommendations. This is particularly problematic in diabetes care, where treatment decisions require transparency to align with patient-specific comorbidities and socioeconomic contexts. While AI-enabled risk stratification tools can identify high-risk patients using variables like waist circumference and BMI, their inability to account for nuanced social determinants of health may perpetuate inequities (246). Furthermore, the lack of standardized regulatory frameworks for AI in healthcare complicates validation and adoption, as evidenced by the reluctance of providers to embrace digital twins (DTs) for personalized treatment simulations due to concerns about data privacy and algorithmic accountability (62).

Methodological challenges also arise from biased training datasets that underrepresent marginalized populations, leading to skewed predictions. AI models trained predominantly on data from high-income countries may fail to generalize to low-resource settings, where diabetes prevalence and complications diverge significantly (247). Unsupervised learning techniques have been proposed to mitigate this by identifying neglected subpopulations (248), but their clinical utility remains unproven. Additionally, conflicting evidence on AI's comparative efficacy complicates its integration: while some studies suggest AI outperforms clinicians in tasks like DR diagnosis, others argue human expertise remains superior for complex cases requiring contextual judgment (244).

Ethical and operational barriers further impede progress. Patient privacy concerns, particularly with wearable technologies that continuously monitor sensitive health data, pose risks of misuse or breaches (247). The high cost of AI-enabled devices exacerbates inequities, as seen in China, where diabetes management is already hampered by disparities in healthcare access (247). Moreover, clinician resistance rooted in inadequate AI literacy and workflow disruptions underscores the need for tailored training programs and sociotechnical integration strategies (246).

Future research must prioritize four key areas: (1) developing FL frameworks to enhance data diversity while preserving privacy (245); (2) advancing XAI techniques to bridge the interpretability gap; (3) establishing equity-focused validation protocols to ensure algorithmic fairness across demographics; and (4) fostering interdisciplinary collaboration among clinicians, data scientists, and policymakers to address sociotechnical barriers. The promise of AI in diabetes care is undeniable—from non-invasive hypoglycemia detection to personalized nutrition apps—but its equitable realization demands rigorous attention to these open problems. Without systemic solutions, AI risks exacerbating rather than alleviating the global diabetes burden (247).

When incorporating federated learning (FL) architectures into AI-based multimodal data streams for precision-equitable diabetes care, the suitability of cross-silo vs. cross-device FL paradigms must be carefully taken into account. Cross-silo FL, which is typified by fewer resource-rich clients, is particularly appropriate for multimodal diabetes care, where various data types—from genomic data and electronic health records (EHRs) to continuous glucose monitoring (CGM) signals and retinal images—require strong computational resources for local model training (14, 249). In keeping with the requirement for privacy-preserving cooperation among healthcare institutions, this architecture guarantees that sensitive patient data remains within institutional silos and permits the aggregation of model updates to improve global predictive performance (14, 249). Cross-silo FL has demonstrated efficacy in federated multi-input models for Alzheimer's disease classification using MRI and tabular data from several hospitals. For diabetic complications like DR or nephropathy, this framework could be adjusted (249). Cross-device FL, designed for scenarios involving thousands of edge devices, is less suitable for multimodal diabetes data due to limitations on bandwidth, processing power, and data heterogeneity. However, by combining lightweight models trained on spectrogram images of CGM signals, it could be helpful for real-time glucose monitoring using wearable sensors, where hypoglycemic events could be identified without endangering personal privacy (250, 251).

Which of these architectures is optimal depends on the trade-offs between clinical utility, resource availability, and data granularity. Cross-silo FL excels in situations requiring high-dimensional data fusion, such as integrating retinal fundus images with EHRs for DR risk stratification, when organizations like tertiary care centers can contribute curated datasets (249). This approach reduces the non-IID (non-independent and identically distributed) data issue that is commonly seen in the healthcare sector by allowing organizations to standardize local data distributions before aggregation (249). The inherent variation in sensor quality and sampling rates among devices makes cross-device FL challenging to manage multimodal data streams despite its scalability (251). However, the volatility of deep learning models like MLPs in cross-device settings, as shown by performance degradation in non-IID scenarios, highlights the need for trustworthy aggregation algorithms appropriate for multimodal data (252).

Innovative FL architectures must also address the integration of conventional statistical techniques, such as multistate models or logistic regression, with AI-driven approaches in order to enhance interpretability and clinical trust. Explainable AI (XAI) frameworks such as SHAP (Shapley Additive Explanations) or LIME (Local Interpretable Model-agnostic Explanations) can bridge this gap by providing clinicians with clear decision-making pathways (62, 253). XAI4Diabetes and DeepNetX2 have demonstrated how interpretable models can elucidate the role of multimodal features in diabetes progression predictions to promote clinician adoption (62, 253). Additionally, federated analytics, a companion to FL, can help harmonize data across silos by combining summary statistics without exchanging raw data, enabling organizations to align disparate data streams for global model training (254, 255). Precision-equitable care depends on ensuring that underrepresented populations benefit from federated insights without increasing biases (253).

Socioeconomic and regulatory factors also have an impact on the choice of FL architecture. FL complies with GDPR and HIPAA through inter-silo Federated learning for DR screening, in which institutions such as the UK Biobank and ADNI (Alzheimer's Disease Neuroimaging Initiative) collaborate to train models without exchanging data (62). Blockchain-secured aggregation, as proposed in decentralized FL (DFL), guarantees model verifiability and lowers single-point-of-failure risks by incorporating an auditable layer into cross-silo workflows (254). On the other hand, in low-resource environments, where wearable-based monitoring prioritizes energy-efficient algorithms like BiLSTM or lightweight CNNs, cross-device FL must manage hardware limitations (251, 256). For DDoS attack detection in IoMT networks, the suggested semi-decentralized FL model—a framework adaptable to diabetes care—offers a compromise by balancing scalability and privacy preservation by clustering IoT devices to minimize communication overhead (256).

The issue of non-independent and identically distributed (non-IID) data in federated learning (FL) is critical in healthcare applications, where real-world datasets display inherent heterogeneity due to variations in patient demographics, clinical practices, and regional healthcare policies (257, 258). This heterogeneity undermines the fundamental tenet of FL, which is that local data distributions are representative of the global population. This leads to unfair client outcomes, poor generalization performance, and biased model convergence (259, 260). Non-IID data in multicenter medical studies may arise from variations in diagnostic criteria, imaging protocols, or disease prevalence, as shown in federated models for detecting delayed cerebral ischemia (DCI), where performance varied significantly across hospitals due to divergent feature distributions and clinical definitions (257). Multilingual FL for depression detection also highlighted the challenges of non-IID data when clients have monolingual datasets with different label distributions, exacerbating imbalances and distorting global model predictions (261).

To solve these problems, creative algorithmic and architectural solutions have been created. One method that lessens feature bias is to separate representation learning from classification using personalized FL frameworks, such as Fed-RepPer. Fed-RepPer uses supervised contrastive loss for local representation learning and combines these into a global model to enable client-specific classifiers and achieve strong performance on non-IID data (262). Another innovation that lessens intra-cluster heterogeneity is clustered FL, which groups clients with similar data distributions. Federated subpopulation models trained on severe DCI cases improved performance for sites with homogeneous severe cohorts, demonstrating the value of targeted aggregation (257). Theoretical developments like weighted local Rademacher complexity, which provide sharper excess risk bounds for non-IID FL, allow frameworks like FedALRC to outperform FedAvg and FedProx by optimizing convergence rates without additional communication overhead (259).

Other strategies to lessen non-IID effects include privacy-preserving enhancements and adaptive aggregation techniques. By employing iterative layer-wise aggregation to address the mismatch between local and global batch normalization parameters—a significant contributor to gradient deviation in non-IID settings—FedTAN achieves strong performance across a range of data distributions (260). Homomorphic encryption and differential privacy (DP) are further integrated to protect sensitive healthcare data while controlling heterogeneity; however, DP's noise addition may inadvertently exacerbate non-IID issues by masking local data patterns (258, 263). Metadata-driven techniques like MetaFedCBT, which employ domain statistics to generate representative brain connectivity templates from decentralized, non-IID neuroimaging data, enhance the global model's centrality and generalizability (264).

In FL, non-IID data has broader implications for fairness and scalability. Label distribution skew and data scarcity can disproportionately disadvantage clients with rare conditions or small datasets, as shown in FL for electronic health records (EHRs), where PEARL's self-supervised learning and fine-tuning scheme improved fairness by tailoring global models to local EHR distributions (263). Communication-efficient methods, such as low-precision decentralized training with Range-EvoNorm, further optimize resource use while preserving accuracy under non-IID conditions and reduce compute energy (265). Fundamental trade-offs still exist, though: while personalized models and clustered FL improve performance, they may also increase complexity or jeopardize global model cohesion (257, 262).

## 5 Future directions

### 5.1 Large medical models

The use of AI in diabetes treatment has the potential to completely transform the field thanks to developments in DT technology, multimodal large models, and human-machine co-governance. These developments address the rising global burden of diabetes and its related complications by offering revolutionary potential in real-time monitoring, personalized diabetes management, and predictive analytics (10, 266). This change is being led by multimodal large models, which combine various data sources like clinical, omics, and lifestyle data. Clinicians can now predict the course of diseases and customize interventions with previously unheard-of precision thanks to these models' ability to synthesize complex datasets using machine learning algorithms (214, 267). AI-powered predictive models have proven effective in predicting the course of diabetic kidney disease (DKD), identifying patients at risk for hemodialysis with 71% accuracy (114). This feature highlights how AI can improve early intervention techniques, which will ultimately lower healthcare costs and improve patient outcomes (8).

The use of AI in diabetes treatment has the potential to revolutionize patient management and healthcare delivery, especially when applied to multimodal large models. The accuracy, effectiveness, and personalization of diabetes care could be greatly improved by these models, which incorporate a variety of data types, including clinical, molecular, and lifestyle data (128, 214). Multimodal large models can handle the intricate, nonlinear relationships present in diabetes pathophysiology by utilizing cutting-edge AI techniques like deep learning and ensemble methods. This provides predictive insights that go beyond conventional linear and logistic regression approaches (8, 114). Studies have demonstrated neural networks can accurately predict the onset and complications of diabetes, demonstrating their generalizability across a wide range of patient populations (52, 268). Early intervention and individualized treatment plans are made possible by the creation of dynamic models that capture temporal patterns and trends through the integration of time-series data, such as CGM and EMR (119, 209).

The use of multimodal large models to integrate heterogeneous data sources, such as wearable technology, imaging, and genomic data, is among the most important developments in this field. This all-encompassing strategy makes it possible to fully comprehend each person's risk factors and the course of the disease, allowing for customized interventions that address cardiovascular and metabolic comorbidities (10, 269). The risk of DKD progression has been evaluated using AI-driven predictive models, which have a 71% accuracy rate in forecasting the start of hemodialysis over a ten-year period (38). AI has demonstrated promise in identifying high-risk individuals and optimizing preventive measures in cardiovascular risk assessment for patients with T2D (39, 41). These developments highlight how multimodal large models can fill gaps in existing therapeutic and diagnostic strategies, providing a more patient-centered and sophisticated framework for diabetes care.

The capacity to process and analyze large datasets with high computational efficiency, revealing hidden patterns and correlations that may be missed by conventional methods, is the technological advantage of multimodal large models. For example, by combining logistic regression analysis and convolutional autoencoders, 3,073 features have been extracted from EMR data, making it possible to predict DKD aggravation with exceptional accuracy (43). Additionally, the use of ensemble models—like those optimized with the Adam algorithm—has shown superior predictive performance, with diabetes onset prediction achieving a ROC AUC value of 0.934 (44). In addition to improving diagnostic precision, these models also improve calibration, making predictions that can be understood and used in clinical settings (146). By incorporating sophisticated neural network architectures, like LSTM models, longitudinal data can be analyzed to gain insights into how diseases progress and how well treatments work over time (145).

Multimodal large models are useful for population-level health management in addition to individual patient care. These models can lessen the financial burden of diabetes and increase the effectiveness of healthcare delivery by identifying high-risk cohorts and allocating resources optimally (147, 148). High negative predictive values have been shown by AI-based DR screening systems, allowing for early detection and intervention in environments with limited resources (51). Patients are empowered to actively participate in their disease management through real-time monitoring and feedback made possible by the integration of AI with wearable technology and telehealth. The emergence of AI-driven personalized mobile healthcare platforms that use DT technology to model patient-specific outcomes and inform treatment choices is an example of this paradigm shift towards digital health and precision medicine.

### 5.2 Digital twin

A virtual depiction of a patient's condition called a DT is another essential component of AI-enabled diabetes treatment. DTs make it easier to continuously monitor, simulate, and optimize treatment plans by building a dynamic, real-time recreation of a patient's physiological state (267). AI-driven recommendations on insulin dosage and lifestyle changes are made possible by integrating insulin pump data, continuous glucose monitoring, and lifestyle variables into a DT framework (10). In addition to empowering people to actively manage their health, this patient-centric approach allows healthcare providers to provide individualized care at scale (266).

The creation of patient-centric DT frameworks based on personal health knowledge graphs (PHKGs) is one of the major advancements in this field. These frameworks combine information from various sources, such as lifestyle data, insulin pump data, and continuous glucose monitoring, to produce a dynamic and thorough depiction of a patient's health (266). Over time, PHKGs improve the precision and accuracy of health insights by facilitating the smooth integration of new patient data. This flexibility is essential for managing diabetes because lifestyle, environmental, and genetic factors can greatly affect how each person reacts to treatment (270). Healthcare professionals can provide focused interventions and preventive measures by using DTs that use AI algorithms to model the course of diseases, forecast treatment outcomes, and perform individualized risk assessments (271).

DTs have proven to be useful in the treatment of diabetes, according to closed-loop trials. To address one of the most difficult aspects of managing diabetes, for example, recent research has used DTs to predict blood glucose levels and optimize insulin dosage in real-time (270). DTs can offer practical insights that enhance glycemic control and lower the risk of complications, according to these trials. Furthermore, DTs have been used to present health information and provide tailored lifestyle advice, enabling patients to take an active role in their treatment (266). DTs' capabilities are further enhanced by the integration of wearable sensors and Internet of Things (IoT) devices, which allow for remote consultations and continuous monitoring—both of which are especially helpful in the management of chronic conditions like diabetes (272).

DTs are also being used to optimize clinical trials and drug development in the treatment of diabetes. DTs expedite the drug discovery process, lower costs, and hasten the release of new treatments by generating virtual patient populations and mimicking treatment outcomes (38). This strategy is especially pertinent to diabetes, where the creation of individualized therapies is crucial to addressing the disease's heterogeneity (39). Additionally, DTs make it easier to find biomarkers and indicators of how a disease will progress, allowing for early detection and treatment (41).

DTs create dynamic, patient-specific models by integrating multimodal data, such as CGM, insulin pump metrics, dietary intake, and activity logs, into virtual replicas of patients (266). By utilizing AI and ML to forecast glycemic fluctuations, optimize insulin dosage, and tailor lifestyle recommendations in real time, these models tackle the interindividual variability that undermines traditional “one-size-fits-all” approaches (273, 274). In addition to improving insulin resistance markers and *β*-cell function, DT platforms have demonstrated significant reductions in HbA1c (−1.8%) and anti-diabetic medication use (−1.5 medications per patient) over the course of a year (274). The bidirectional feedback loop of DTs enables them to continuously align their simulations with patient outcomes while also assimilating real-world data, thereby enabling adaptive precision medicine (10).

The ability of DTs to operationalize personal health knowledge graphs (PHKGs), which organize diverse data into useful insights, is a crucial benefit (266). PHKGs increase equity by ensuring that disadvantaged groups receive targeted, context-aware interventions, such as considering food insecurity or cultural dietary preferences in glycemic management (273). In terms of incremental glucose control, algorithms such as PNP outperform static dietary guidelines by 16.73% when using gut microbiome data to predict postprandial glucose responses (PPGRs) (273). Additionally, by incorporating telehealth, DTs close gaps in underserved or rural communities through remote monitoring and nudges (274). However, there are still ethical conundrums, such as risks to data privacy and algorithmic biases that could exacerbate inequality if training datasets lack diversity (270). Robust governance frameworks are essential to ensure DTs adhere to HL7 standards for interoperability while safeguarding patient autonomy (275).

Beyond clinical care, DTs have translational potential in the fields of public health and drug development. By accelerating clinical trials, simulating virtual patient cohorts, and predicting therapeutic efficacy and adverse events before actual testing, DTs lower costs and ethical burdens (276). Comparable DT frameworks have enabled respiratory medicine to forecast the disease's progression, indicating comparable uses for diabetes complications (277). As demonstrated by Human Digital Twin (HDT) prototypes for elderly T2D patients, which decreased hypoglycemic events by 45% through adaptive MPC-based insulin delivery, future directions include hybrid modeling (combining physics-based and data-driven approaches) to improve predictive accuracy for comorbid conditions (278). Scalability is still a challenge, requiring federated learning and cloud-based architectures to deploy DTs across healthcare systems without sacrificing data security (279, 280).

### 5.3 Human-machine co-governance

The concept of human-machine co-governance, which stresses cooperation between AI systems and medical practitioners, is essential to optimizing AI's advantages in diabetes treatment. Although AI is very good at processing data and identifying patterns, human knowledge is essential for contextualizing AI results and making complex medical decisions (146). AI recommendations have been demonstrated to perform better than physician decisions in virtual urgent care settings when it comes to spotting important warning signs and following clinical guidelines, but doctors are still better at tailoring recommendations to the needs of specific patients (146). When handling complex cases, this synergy between AI and human judgment is especially beneficial because AI can be used as a decision-support tool rather than as a substitute for clinical expertise (147). Establishing trust and guaranteeing fair access to AI-driven healthcare solutions also requires addressing the ethical implications of AI, such as data privacy and algorithmic transparency (51).

Algorithmic bias presents unique challenges in FL settings due to decentralized data silos, heterogeneous distributions, and privacy-preserving constraints. To quantify and reduce such biases, novel approaches that balance fairness with the inherent limitations of FL are required. Intersectionality theory-based differential fairness (DF) metrics have been adapted to FL to measure differences across overlapping protected attributes without requiring centralized data access (281). Even with sparse or dispersed data, these metrics assess fairness by evaluating parity in error rates across subgroups (281, 282). In healthcare FL applications, such as forecasting 30-day readmissions, fairness drift—where bias manifests after deployment—has been observed. This necessitates continuous monitoring to find variations in calibration or accuracy across racial or socioeconomic groups using metrics like the generalized entropy index and equalized odds difference (282, 283).

Common mitigation strategies in FL include local debiasing and fairness-aware model aggregation. The Capuchin algorithm uses adversarial debiasing during federated updates, where local models are trained to lessen bias amplification while retaining utility (284). Similarly, Fairlearn has been extended to FL settings so that users can collaboratively optimize for fairness constraints through iterative reweighting of local gradients (284). Post-processing techniques like equalized odds adjustment are also applied to federated outputs in order to mitigate biases in predictions for underrepresented groups (284). Empirical studies show that incorporating race-interaction terms into FL models improves fairness for non-White subgroups by reducing misclassification rates in EHR-continuity predictions by two to three times (285).

In order to align ethical norms with technological debiasing, the SHIFT paradigm highlights the social implications of FL bias and encourages interdisciplinary collaboration (286). Participatory FL frameworks ensure that models reflect a range of care scenarios by incorporating stakeholders in the co-design of fairness restrictions (287). This is operationalized by tools such as D-BIAS, which visualize causal relationships in federated data and enable users to simulate debiased datasets with minimal distortion and iteratively refine biased edges (288).

Data heterogeneity in FL can exacerbate bias, as shown in sleep-scoring algorithms where age-related biases emerged due to unequal training distributions (289). Two strategies to balance subgroup representation are federated reweighting and synthetic data augmentation (288). The NIST AI Bill of Rights and the GDPR's risk-based approach mandate that FL systems document bias mitigation strategies, such as pre-market evaluations for high-risk medical devices (290).

Federated settings need cooperative debiasing techniques, context-aware fairness metrics, and stringent governance to ensure equitable AI. By combining intersectional fairness measures, adversarial training, and stakeholder feedback, FL can advance toward bias-resistant models without compromising privacy or usefulness.

The integration of clinicians, patients, and community stakeholders in the development, validation, and application of human-machine co-governance models—which prioritize ethical accountability and participatory design—represents the TA paradigm shift in healthcare technology. This approach addresses the critical tension between societal benefits and individual risks, particularly in circumstances such as COVID-19 vaccination where model results impact critical decisions (291). Although population-level models may prioritize aggregate outcomes, individual stakeholders, such as remote workers in low-incidence areas, may rationally decide not to participate in interventions like the AZ vaccine due to individualized risk assessments. This divergence emphasizes the need for inclusive modeling frameworks that take into account a range of value judgments, as evidenced by initiatives like ISPOR's recommendations for involving clinicians, legislators, and patients in model development. However, there are still gaps in practice; many models were created without formal stakeholder collaboration, which puts marginalized voices at risk of epistemic injustices (291).

The multidisciplinary lifecycle management for AI/ML tools at Duke Health is an example of an innovative governance framework that shows how structured engagement can reduce biases and improve practical applicability (292). These frameworks guarantee that models are in line with clinical workflows while addressing equity concerns, such as avoiding algorithmic biases that disproportionately harm vulnerable populations, by combining regulatory best practices with ongoing monitoring (292). In a similar vein, The Lancet-FT Commission's participatory approaches to digital health governance prioritize community enfranchisement as a means of fostering trust and facilitating democratic oversight and equitable access (293). There are still gaps in practice, though, as many models were developed without formal stakeholder collaboration, putting marginalized voices at risk of epistemic injustices.

Human-machine co-governance also requires reconsidering traditional hierarchies and shifting toward inclusive modalities that emphasize transparency and cooperative decision-making (294). Levinasian ethics highlights the tension between distributive justice and relational ethics by urging models to find a balance between measurable outcomes and individualized care (295). Technologies like explainable AI (XAI) and dynamic consent mechanisms can bridge this gap by empowering patients to co-manage data use and algorithmic outputs. Self-determined data sharing is made possible by patient-controlled “knowledge commons” in cystic fibrosis care models (296). These systems address issues of enclosure and disempowerment while also adhering to GDPR regulations by granting patients control over their health data (296).

The operationalization of co-governance is hampered, though, particularly when it comes to striking a balance between technical and human factors. Human-in-the-loop (HITL) approaches are robust in psychiatric applications, but they are still limited by dataset quality and require safety measures like diversity sampling and reliability metrics to prevent inaccurate predictions (297). The lack of Global South perspectives in health AI governance and the underrepresentation of AI developers in legal-risk discussions are two examples of how disciplinary silos frequently obstruct interdisciplinary collaboration (298). Suggested solutions include institutionalized norms for public participation, akin to engineering safety standards, and adaptive model architectures that integrate real-time feedback from patients and clinicians (294). Task and theory-of-mind models in HITL systems must be updated frequently to reflect evolving clinical scenarios and ergonomic requirements in order to guarantee alignment with human cognitive and physical constraints (299).

The future of human-machine co-governance will ultimately depend on systemic approaches that integrate emancipatory goals—like equity and trust—into AI design and implementation. This necessitates both structural and technological changes, such as rules requiring providers to be AI literate and roundtable forums for stakeholder discussions (294, 296). These models can go beyond neoliberal efficiency paradigms to provide patient-centered, ethically sound care by elevating marginalized voices and encouraging interdisciplinary discourse (295, 298).

Future prospects for improving diabetes care are extremely bright thanks to the convergence of DTs, multimodal large models, and human-machine co-governance. Future studies should concentrate on creating strong AI algorithms that can reason causally, incorporating data from various populations in the real world, and confirming these technologies through extensive clinical trials (267). A comprehensive, patient-centered approach to healthcare may be made possible by extending DT frameworks to include additional chronic conditions (266). Overcoming technological, moral, and legal obstacles will require promoting interdisciplinary cooperation between computer scientists, physicians, and legislators (8). The application of AI to diabetes care has the potential to revolutionize healthcare and usher in a period of precision medicine and proactive disease management, in addition to improving clinical outcomes as the technology develops (267).

## 6 Conclusion

AI has proven to be a valuable tool in diabetes care, improving patient outcomes, personalizing treatment plans, and increasing diagnostic accuracy. In order to minimize complications and medical expenses, AI-driven algorithms have demonstrated efficacy in identifying glycemic episodes, forecasting disease progression, and refining treatment plans. Real-time monitoring and remote patient management have been transformed by the combination of AI with wearable technology and telehealth platforms, providing previously unheard-of convenience and accuracy in the treatment of diabetes. These developments highlight AI's revolutionary potential in tackling the worldwide burden of diabetes, a chronic illness with rising incidence and financial consequences.

Harnessing the full potential of AI in diabetes care requires interdisciplinary collaboration between healthcare providers, technologists, policymakers, and industry stakeholders. Collaborations across industries can spur AI algorithm development and guarantee that these algorithms can be tailored to a range of patient types and healthcare environments. Furthermore, in order to address ethical issues like algorithmic bias and data privacy and promote openness and confidence in AI-driven interventions, regulatory frameworks must change. Standardizing AI applications, encouraging fair access, and incorporating human oversight to reduce risks and errors are all critical tasks for policymakers. Stakeholders can jointly promote the moral and efficient application of AI in diabetes care by cultivating a cooperative ecosystem, which will ultimately improve patient outcomes and lessen the cost of healthcare worldwide.

The unrealized potential of AI in diabetes care must be investigated by researchers, physicians, business executives, and regulators working together to solve current issues and spur innovation for a healthier future. Through the prioritization of ethical considerations and interdisciplinary collaboration, the healthcare community can guarantee that AI technologies are not only patient-centered and socially responsible, but also scientifically sound.
